# Repurposing cepharanthine as a radiosensitizer in esophageal squamous cell carcinoma through dual metabolic intervention and direct targeting of p70s6K

**DOI:** 10.1186/s12967-026-08550-y

**Published:** 2026-07-03

**Authors:** Zhihua Hao, Xin Su, Jiantao Li, Jing Jin, Zheng Li, Zhiqiang Yan, Wenpeng Jiao, Wenjing Jiao, Yingying Wang, Chenguang Ji, Xuan Wang, Yutong He

**Affiliations:** 1 Cancer Institute in Hebei Province, The Fourth Hospital of Hebei Medical University, Shijiazhuang, 050000 China; 2 School of Public Health, Hebei Medical University, Shijiazhuang, Hebei 050000 China; 3 Scientific Research Center, The Second Hospital of Hebei Medical University, Shijiazhuang, Hebei 050000 China; 4 Department of Radiation Oncology, Hebei General Hospital, Shijiazhuang, Hebei 050000 China; 5 Department of Radiation Oncology, The Fourth Hospital of Hebei Medical University, Shijiazhuang, Hebei 050000 China; 6 Clinical Laboratory, The Fourth Hospital of Hebei Medical University, Shijiazhuang, Hebei 050000 China; 7 Department of Gastroenterology, The Second Hospital of Hebei Medical University, Shijiazhuang, Hebei 050000 China

**Keywords:** Esophageal squamous cell carcinoma, Radioresistance, Metabolic duality, Cepharanthine, p70S6K, Radiosensitizer, Ubiquitination

## Abstract

**Background:**

Metabolic reprogramming underpins the acquisition of radioresistance in esophageal squamous cell carcinoma (ESCC); however, the specific bioenergetic vulnerabilities and direct pharmacological targets remain to be fully elucidated. This study defines a distinct metabolic phenotype conferring radioresistance and evaluates the natural alkaloid Cepharanthine (CEP) as a mechanism-driven radiosensitizer.

**Methods:**

Matched clinical cohorts of radiosensitive and radioresistant ESCC patients were analyzed using widely-targeted and targeted metabolomics. Bioenergetic profiling (ECAR/OCR) was performed on established isogenic radioresistant cells. The mechanistic interactions between CEP and its target were mapped via network pharmacology, surface plasmon resonance (SPR), cellular thermal shift assays (CETSA), ubiquitin-proteasomal degradation assays, and Q347A site-directed mutagenesis. In vivo efficacy was validated across human cell-derived xenografts (CDX) and immunocompetent syngeneic (AKR/C57BL/6) mouse models.

**Results:**

Clinical multi-omics revealed a "metabolic duality" in radioresistant ESCC, characterized by the concurrent hyperactivation of glycolysis and oxidative phosphorylation (OXPHOS). CEP administration disrupted this metabolic network, significantly sensitizing ESCC cells to irradiation [Dose-modifying factor at 37% survival (DMF_37_) > 1]. Mechanistically, CEP directly engages the kinase domain of p70S6K—a structural interaction dependent on the Q347 residue—and triggers its ubiquitin-proteasomal degradation. This targeted clearance disrupts the upstream PI3K/Akt/mTOR survival axis. Genetic overexpression of wild-type p70S6K, but not the Q347A mutant, rescued the dual hypermetabolic phenotype and reinstated radioresistance. Clinically, elevated p70S6K expression correlated with poor disease-free survival and therapeutic failure. In vivo, CEP synergized with radiotherapy to suppress tumor kinetics in both CDX and syngeneic models, while concurrently enhancing CD8^+^ T cell infiltration in the immunocompetent microenvironment, with no observable systemic toxicity.

**Conclusions:**

Radioresistant ESCC relies on a dual hypermetabolic state driven by the PI3K/Akt/mTOR/p70S6K cascade. CEP overcomes this radioresistance by physically binding to and degrading p70S6K, thereby inducing bioenergetic exhaustion and reshaping the anti-tumor microenvironment. These findings provide a solid mechanistic rationale for translating CEP into clinical radiotherapeutic regimens.

**Supplementary Information:**

The online version contains supplementary material available at 10.1186/s12967-026-08550-y.

## Introduction

Esophageal squamous cell carcinoma (ESCC) represents the predominant histological subtype of esophageal cancer, accounting for the vast majority of cases in endemic regions [1, 2]. Although radiotherapy constitutes a fundamental pillar in the clinical management of ESCC, its therapeutic efficacy is frequently compromised by intrinsic or acquired radioresistance, leading to local recurrence and subsequent treatment failure [3]. The underlying mechanisms driving this resistance are multifaceted. Emerging evidence highlights metabolic reprogramming not merely as a byproduct of oncogenesis, but as a critical adaptive survival strategy [4]. While the Warburg effect (aerobic glycolysis) has long been considered a hallmark of tumor metabolism [5], recent studies indicate that radioresistant cells exhibit metabolic plasticity [6, 7]. Specifically, these cells can acquire a phenotype of “metabolic duality” — the concurrent hyperactivation of both aerobic glycolysis for macromolecular biosynthesis and oxidative phosphorylation (OXPHOS) for efficient ATP production. This dual energy dependency provides a robust survival advantage under the oxidative stress induced by ionizing radiation [8–10].

Disrupting this adaptive metabolic network represents a promising strategy to overcome radioresistance. Consequently, there is an urgent clinical need to identify safe and potent metabolic modulators. Cepharanthine (CEP), a natural bisbenzylisoquinoline alkaloid, possesses well-documented anti-tumor properties and a favorable biosafety profile [11, 12]. While traditionally recognized for its ability to reverse multidrug resistance, recent studies suggest that CEP modulates central oncogenic signaling networks. Within the context of metabolic regulation, the PI3K/Akt/mTOR cascade is frequently hyperactivated in ESCC and is a key driver of radioresistance [13, 14]. As a primary downstream effector of this axis, p70S6K acts as a critical signal integrator, translating mitogenic stimuli into metabolic reprogramming [15]. However, the precise structural mechanism by which pharmacological agents might target p70S6K to dismantle the dual metabolic phenotype in ESCC remains largely undefined.

In the present study, we hypothesized that CEP functions as a metabolic radiosensitizer by directly targeting the p70S6K-driven bioenergetic network. Utilizing matched clinical cohorts, we first established that radioresistant ESCC patients exhibit a distinct dual hypermetabolic state. We subsequently identified CEP as a potent disruptor of this phenotype. Mechanistically, we demonstrate that CEP physically binds to the Q347 residue of the p70S6K kinase domain, triggering its ubiquitin-proteasomal degradation and thereby causing severe metabolic dysfunction. Furthermore, utilizing both immunodeficient xenografts and immunocompetent syngeneic mouse models, we confirmed that CEP exerts synergistic radiosensitizing effects in vivo, which is accompanied by enhanced CD8^+^ T cell infiltration in the tumor microenvironment. Collectively, our findings define metabolic duality as a targetable vulnerability in radioresistant ESCC and provide a structural and functional rationale for translating CEP into clinical radiotherapeutic regimens.

## Materials and methods

### Ethics statement

All human studies received approval from the Ethics Committee of the Fourth Hospital of Hebei Medical University (Approval No. 2023KY027), and written informed consent was obtained from all participants involved. Additionally, the animal experiments adhered to the ARRIVE guidelines and were approved by the Institutional Animal Welfare Committee (Approval No. 2023204 and 20260166).

### Patient cohort and sample collection

A total of 62 patients with histologically confirmed ESCC undergoing radiotherapy at the Fourth Hospital of Hebei Medical University were recruited using a prospective-retrospective design (March–December 2023, Table S1). Inclusion criteria: (1) histological ESCC confirmation; (2) no prior radiotherapy/chemotherapy; (3) tolerance to thoracic radiation of ≥ 36 Gy. Exclusion criteria: (1) prior thoracic surgery; (2) malignant effusions; (3) concurrent malignancies. Tumor clinical responses were classified into radiosensitive (Partial Response, PR; *n* = 31) and radioresistant (Stable Disease/Progressive Disease, SD/PD; *n* = 31) groups at 1 month post-radiotherapy according to RECIST 1.1 guidelines [16]. For metabolomic analysis, a discovery cohort comprising 7 matched PR and SD patient pairs was subjected to widely-targeted Q300 metabolomic profiling (Table S2). Subsequently, the differential bioenergetic signatures were validated in an expanded cohort of 17 independent, clinically baseline-matched patient pairs using targeted energy metabolomics (Table S3). Ten additional surgical ESCC patients (January–June 2023) who did not receive radiotherapy provided tumor/adjacent tissues for immunohistochemistry (IHC) (Table S5).

### Comprehensive and targeted metabolomics

Plasma samples (20 µL) or cell pellets (1 × 10^7^ cells) were extracted using cold methanol containing internal standards. For widely-targeted profiling, samples were derivatized and analyzed on an ultra-performance liquid chromatography-tandem mass spectrometry (UPLC-MS/MS) system. For targeted energy metabolomics, a high-throughput liquid chromatography triple-quadrupole mass spectrometry (LC-MS/MS) platform was employed to monitor specific glycolytic and TCA cycle intermediates. Differential metabolites were identified based on Variable Importance in Projection (VIP) > 1.0 and *P* < 0.05. Pathway enrichment analysis was performed using the Kyoto Encyclopedia of Genes and Genomes (KEGG) database.

### Cell culture and radioresistant subline

The human ESCC cell lines KYSE150, TE-1, and KYSE410 (Zhongqiaoxinzhou Biotech, Shanghai, China) were cultured in RPMI 1640 medium (Gibco, New York, USA), whereas the AKR cell line (Zhongqiaoxinzhou Biotech) was cultured in DMEM. All culture media were supplemented with 10% fetal bovine serum (FBS, Gibco) and 1% penicillin-streptomycin. All cells were maintained in a humidified incubator at 37 °C with 5% CO₂. The radioresistant subline KYSE150R was established by exposing parental cells to nine cycles of 2 Gy X-ray irradiation using an Elekta linear accelerator (Sweden). Radioresistance was confirmed via MTS cell viability and colony formation assays.

### Pharmacological treatments

Cepharanthine (CEP, ≥ 98% purity, Aladdin, Shanghai, China) was dissolved in DMSO and stored at -20 °C. Cells were divided into four groups: Control (untreated); CEP group (at concentrations ranging from 0 to 100 µM for 4 days); IR group (pretreated with medium for 2 days, irradiated 0–10 Gy X-ray irradiation, then cultured for 2 days); IR + CEP group (pretreated with CEP for 2 days, irradiated, then treated with CEP for 2 days).

### Clonogenic survival assay and DMF_37_ calculation

ESCC cells were seeded into 6-well plates at titrated densities (300, 600, 2000, and 4000 cells/well for 0, 2, 4, and 6 Gy, respectively). After 24 h of attachment, cells were treated with a sub-lethal concentration of Cepharanthine (CEP) or vehicle control, followed by ionizing radiation (0, 2, 4, and 6 Gy). After 10 to 14 days of continuous incubation, macroscopic colonies (defined as > 50 cells) were fixed with 4% paraformaldehyde, stained with 0.1% crystal violet, and counted. The survival fraction (SF) was calculated by normalizing to the plating efficiency (PE) of the 0 Gy control group. SF data were mathematically fitted to the linear-quadratic (LQ) model (SF = exp(-αD-βD^2^)) using GraphPad Prism. To quantify radiosensitizing efficacy, the dose-modifying factor at 37% survival (DMF_37_) was calculated as the ratio of the radiation dose required to achieve 37% survival in the control group divided by the equivalent dose for the CEP-treated group. A DMF*37* > 1 defines a radiosensitizing effect.

### Antibodies and reagents

The reagents and antibodies used were as follows: mTOR (1:500, HY-P80231, MedChem Express, Monmouth Junction, NJ, USA), phospho-mTOR (1:500, HY-P80469, MedChem Express), HIF-1α (1:500, HY-P80704, MedChem Express), HK2 (1:1000, #2867, Cell Signaling Technology, Danvers, MA, USA), phospho-p70S6K (1:1000, #9234, Cell Signaling Technology), p70S6K (1:5000, ab32529, Abcam, Cambridge, UK), PI3K (1:1000, ab191606, Abcam), phospho-PI3K (1:1000, #17366, Cell Signaling Technology), PKM2 (1:1000, #4053, Cell Signaling Technology), AKT1 (1:500, HY-P80008, MedChem Express), phospho-AKT1 (1:500, HY-P80788, MedChem Express), β-actin (1:1000, GB11001, Servicebio, Wuhan, China), LDHA (1:5000, 19987-1-AP, Proteintech, Wuhan, China), NQO1 (1:10000, ab80588, Abcam), Cleaved PARP1 (1:500, HY-P80625, MedChem Express), PARP (1:1000, #9542, Cell Signaling Technology), Caspase 3 (1:5000, AB32351, Abcam), Caspase 9 (1:300, 10380-1-AP, Proteintech), RPS6 (1:1000, R012138, Epizyme Biotech, Shanghai, China), phospho-RPS6 (1:1000, R015190, Epizyme Biotech), HA (1:2000, 51064-2-AP, Proteintech), Flag (1:10000, 20543-1-AP, Proteintech), CD8 (1:20000, 66868-1-lg, Proteintech). They were used according to the manufacturer’s instructions.

### Transcriptomics

Libraries were prepared from 1 µg total RNA using the ABclonal mRNA-seq Kit. Sequencing was performed on Illumina NovaSeq 6000/MGISEQ-T7 platforms. Reads were aligned to the reference genome using HISAT2, and gene expression (FPKM) was quantified via FeatureCounts. Differential expression analysis (|log2FC| > 1, Padj < 0.05) used DESeq2. GO term and KEGG enrichment analyses were performed using the clusterProfiler package. Bioinformatics pipelines were developed by Shanghai Applied Protein Technology.

### Cell proliferation assays

For MTS assays, 1,000 cells/well were seeded in 96-well plates, incubated with MTS (G358A, Promega, USA) solution for 2.5 h, and absorbance was measured at 490 nm (Bio-Rad, USA). For colony formation assays, 2,000 cells/well were seeded in 6-well plates, cultured for 10 days before fixing and staining.

### Flow cytometry

Cells were pretreated and analyzed using ROS detection (S0033, Beyotime, Shanghai, China) or Annexin V-FITC apoptosis kits (C1062, Beyotime) according to manufacturer’s protocols. Analysis was performed using a flow cytometer (Beckman, Pasadena, CA, USA).

### Seahorse assay

Extracellular acidification rate (ECAR) and mitochondrial oxygen consumption rate (OCR) were measured using a Seahorse XFe96 Analyzer (Agilent, Palo Alto, CA, USA). Cells were plated in XF media with 10 mM glucose, 2 mM glutamine, and 1 mM sodium pyruvate. ECAR was measured after sequential injection of Rot/AA (0.5 µM) and 2-DG (50 mM). OCR was detected following injection of oligomycin (1.5 µM), FCCP (1.0 µM), and rotenone/antimycin A (Rot/AA) (0.5 µM). All values were normalized to the cell number in each well and plotted as the mean ± SD.

### Western blotting

Proteins were extracted using lysis buffer (G2002, Servicebio), separated by SDS‒PAGE, transferred to PVDF membranes (Millipore, Germany), and probed with primary antibodies overnight at 4 °C. Signals were visualized and normalized to β-actin.

### RT‒qPCR

Total RNA was isolated with TRIzol (15596018CN, Thermo Fisher), reverse-transcribed, and amplified using SYBR Green Master Mix (GLPBIO, USA) on an ABI 7500 system (40 cycles: 95 °C/15 s, 60 °C/1 min). The primer sequences used in this study are listed in Supplementary Table S6.

### ELISA

Plasma p70S6K was quantified using a competitive ELISA kit (SEL979Hu, Cloud-Clone, Wuhan, China) according to the manufacturer’s instructions.

### Immunohistochemistry (IHC) and histological analysis

Formalin-fixed, paraffin-embedded tumor tissues were cut into 5-µm sections. The sections were deparaffinized in xylene and rehydrated through graded ethanol series. Heat-induced antigen retrieval was performed, followed by quenching of endogenous peroxidase activity with 3% H_2_O_2_ and blocking with 5% BSA. The slides were then incubated with primary antibodies (anti-p70S6K, anti-Ki-67, or anti-CD8) overnight at 4 °C. After washing, sections were incubated with an HRP-conjugated secondary antibody. Immunoreactivity was visualized using a DAB substrate kit, and cell nuclei were counterstained with hematoxylin. Following IHC staining, the slides were examined under a light microscope. The relative protein expression levels and tissue distribution patterns of p70S6K, Ki-67, and CD8 were scored semi-quantitatively by two independent, experienced pathologists who were blinded to the experimental treatment groups. Representative high-power fields (HPFs) best reflecting the overall treatment effects were selected per section for image acquisition.

### Targeted metabolic blockade assay

To functionally assess the contribution of metabolic duality to the radioresistant phenotype, targeted metabolic blockade was performed using 2-Deoxy-D-glucose (2-DG, 1 mM) as a glycolysis inhibitor and oligomycin (5 nM) as an OXPHOS inhibitor. Pathway-specific inhibition was confirmed via real-time metabolic flux analysis (ECAR and OCR). For survival evaluations, KYSE150R cells were pretreated with individual or combined metabolic inhibitors prior to irradiation with a fixed dose of 4 Gy. Macroscopic colony formation was subsequently evaluated following the standard clonogenic assay protocol described above.

### TUNEL staining

Apoptosis in tumor sections was assessed using a TUNEL kit (G1501, Servicebio) according to the manufacturer’s protocol.

### Network pharmacology and molecular docking

CEP targets were identified from DGIdb, GeneCards, SwissTargetPrediction, and TargetNet. Protein‒protein interaction (PPI) networks were constructed via STRING and visualized in Cytoscape (v3.9.1). The Molecular Complex Detection (MCODE) plugin in Cytoscape was utilized to extract and identify highly interconnected key subnetwork clusters from the overarching PPI network. Molecular docking with p70S6K (PDB: 3A60) was performed using AutoDock Vina.

### Cellular thermal shift assay (CETSA)

To evaluate the direct physical engagement between CEP and p70S6K, CETSA was performed. Briefly, whole-cell lysates were extracted and incubated with 100 µM CEP or an equivalent volume of DMSO for 1 h. The lysates were equally aliquoted into PCR tubes and subjected to a transient heating gradient (55–85 °C) for 3 min, followed by a 3-min cooling period at room temperature. After high-speed centrifugation (12,000×g, 20 min, 4 °C) to precipitate heat-denatured protein aggregates, the soluble supernatants were collected, boiled with loading buffer, and analyzed via Western blotting using an anti-p70S6K antibody.

### Surface plasmon resonance (SPR) binding assay

The interaction between CEP and p70S6K was analyzed using a Biacore T200 instrument (Cytiva). Recombinant p70S6K (HY-P76588, MCE) was immobilized on a CM5 chip. CEP (0.625–400 µM) was injected at 30 µL/min. Sensorgrams were analyzed using a 1:1 binding model.

### Plasmids, site-directed mutagenesis, and lentiviral transfection

Human wild-type (WT) p70S6K cDNA was cloned into the pLenti-CMV-3FLAG-Puro vector to generate the OE-p70S6K construct. The p70S6K-Q347A point mutant (OE-Q347A) was generated using the QuikChange Site-Directed Mutagenesis Kit with specific primers modifying the Q347 codon to alanine and confirmed by Sanger sequencing (Table S6). For lentiviral packaging, HEK293T cells were co-transfected with the respective lentiviral vectors and helper plasmids (psPAX2 and pMD2.G) using Lipofectamine 3000. Stable ESCC sublines (KYSE150 and KYSE150R) overexpressing WT or Q347A p70S6K were established via viral transduction and selected with 2 µg/mL puromycin for 7 days.

### Cell-based ubiquitination and cycloheximide chase assays

For protein stability analysis, cells were treated with 50 µg/mL cycloheximide (CHX; Sigma-Aldrich) for the indicated durations (0, 2, 4, 8, 12 h) in the presence or absence of CEP (10 µM), followed by Western blot analysis. For the cell-based ubiquitination assay, cells were transiently co-transfected with pLenti-3FLAG-p70S6K and HA-tagged ubiquitin (HA-Ub) plasmids. At 48 h post-transfection, cells were exposed to CEP (10 µM) for 6 h, with the addition of the proteasome inhibitor MG132 (10 µM; MedChemExpress) for the final 4 h to prevent target degradation. Whole-cell lysates were harvested in RIPA buffer, immunoprecipitated with an anti-p70S6K antibody or anti-FLAG magnetic beads overnight at 4 °C, washed, eluted, and subjected to immunoblotting with an anti-HA antibody to visualize polyubiquitinated p70S6K species.

### In vivo xenograft and syngeneic studies

To evaluate the translational potential of CEP, an animal study was initiated utilizing 20 male C57BL/6 mice and 40 immunodeficient BALB/c nude mice (4 weeks of age). The immunocompetent syngeneic model was established by subcutaneously injecting AKR mouse ESCC cells into the right flank of the C57BL/6 mice. The nude mice were utilized to establish human cell-derived xenograft (CDX) models (KYSE150 and KYSE150R).

When tumors reached a palpable volume of approximately 100 mm³, the mice were randomized into four designated treatment cohorts: Control (vehicle), CEP, IR, and IR + CEP. Radiotherapy (IR) was administered using an Elekta linear accelerator equipped with a customized lead shield. The localized X-ray irradiation was delivered as a fractionated dose of 12 Gy total (6 Gy/fraction, 2 fractions) for both the human cell-derived xenograft (CDX) models and the syngeneic model. CEP was administered daily via oral gavage for 21 consecutive days. To match the distinct pharmacokinetic tolerance of the C57BL/6 mice, the CEP dosage was optimized to 20 mg/kg/day for the AKR model, compared to 15 mg/kg/day for the xenograft models.

Tumor volumes (calculated as 0.5 × length × width²) and mouse body weights were closely monitored and recorded every 3 days. On Day 21 (xenografts) or Day 15 (AKR model), the mice were humanely euthanized. Excised tumors and major vital organs (heart, liver, spleen, lungs, and kidneys) were harvested and processed for downstream histological evaluation (H&E, Ki-67, p70S6K, CD8, TUNEL) and Western blot analyses.

### Statistical analyses

Data are presented as means ± SD (*n* ≥ 3). Statistical significance was determined using Student’s t-test (two-group comparisons), one-way ANOVA (multi-group comparisons), or Fisher’s exact test (categorical data) in SPSS v26.0. For one-way ANOVA, *post hoc* pairwise comparisons were performed with the Bonferroni method to adjust for multiple testing.

## Results

### Targeted metabolic profiling reveals a dual hypermetabolic state in radioresistant ESCC

**Fig. 1:**
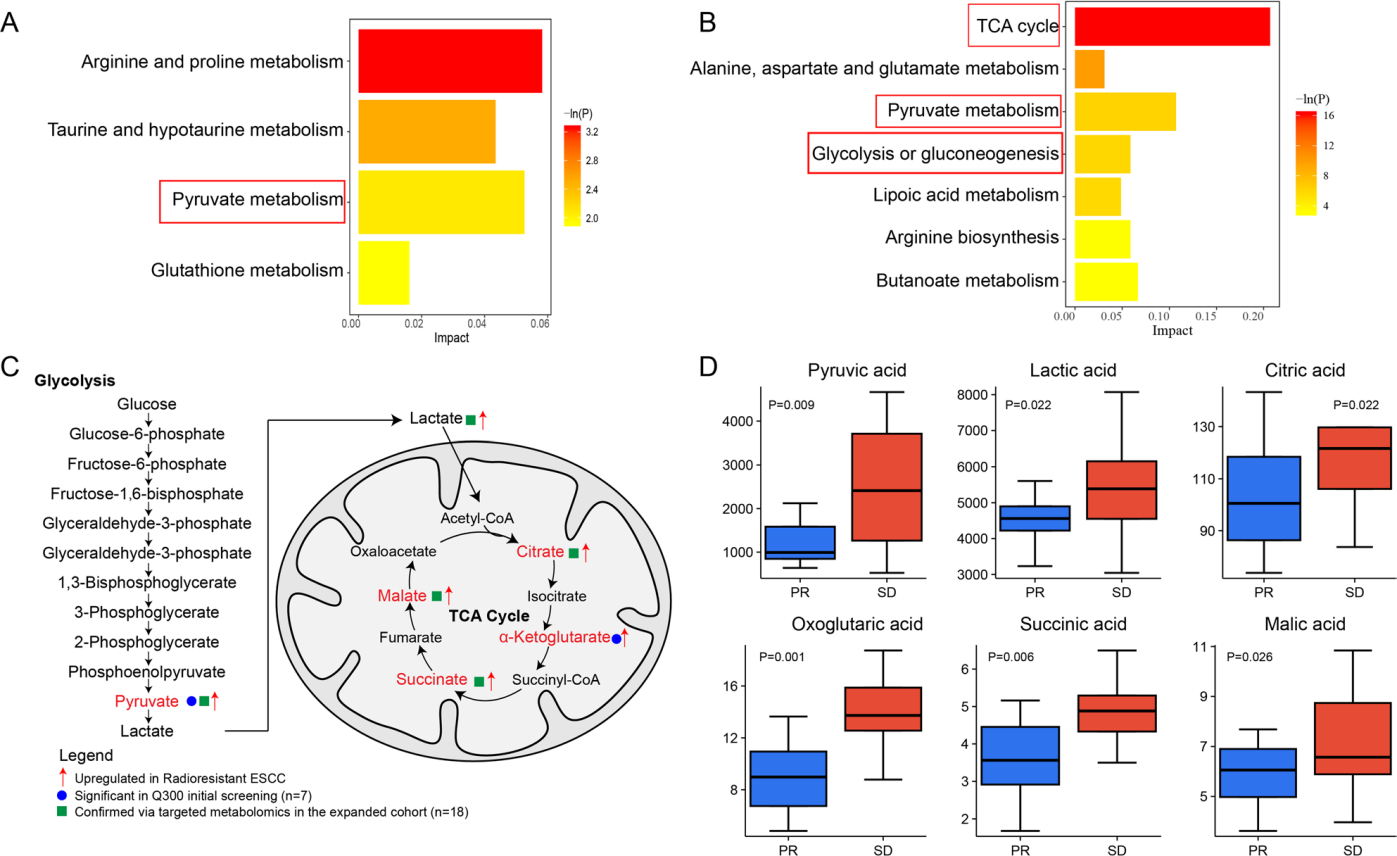
Identification and validation of the metabolic duality signature in radioresistant ESCC patients. **A** KEGG pathway enrichment from widely-targeted metabolomics (Q300) in the discovery cohort (*n* = 7 pairs), highlighting alterations in pyruvate metabolism. **B** KEGG pathway enrichment from targeted metabolomics in the expanded validation cohort (*n* = 17 pairs), confirming the hyperactivation of the TCA cycle and glycolysis. **C** Schematic map of the dual metabolic hyperactivation. Red text indicates significant upregulation in the radioresistant (SD) group. Blue circles and green squares denote significance in the discovery and validation cohorts, respectively. **D** Boxplots of six key upregulated metabolites in the PR vs. SD group within the validation cohort (*n* =17 pairs). Boxes show median and interquartile range. *P*-values were determined by Student's *t*-test or Mann-Whitney U test

Following one-month post-radiotherapy assessments, ESCC patients were classified into radiosensitive (PR, *n* = 31) and radioresistant (SD, *n* = 31) groups (Table S1). We initially subjected matched PR and SD subgroups (*n* = 7 pairs; balanced for TNM stage and baseline characteristics, Table S2) to widely-targeted Q300 metabolomics, which highlighted early alterations in pyruvate metabolism (Fig. 1A).

To validate these findings, we performed targeted energy metabolomics on an expanded cohort of 17 patient pairs (Table S3). This analysis confirmed coordinated shifts in both pyruvate and mitochondrial pathways among radioresistant patients (Fig. 1B), mapping a transition toward concurrent glycolytic and mitochondrial energy production (Fig. 1C). Accordingly, key metabolites driving this dual hypermetabolic state—pyruvic acid, lactic acid, citric acid, oxoglutaric acid (α-KG), succinic acid, and malic acid—were significantly elevated in the expanded SD cohort (Fig. 1D).

A systematic comparison of both cohorts (Table S4) shows that while some TCA cycle intermediates exhibited non-significant upward trends (FC > 1) in the initial screening, their significant upregulation was verified in the expanded cohort. Core metabolites like pyruvic acid and oxoglutaric acid were consistently upregulated throughout. Together, these clinical data establish the dual hyperactivation of glycolysis and mitochondrial respiration in radioresistant ESCC.

### In vitro validation of the dual hypermetabolic phenotype in radioresistant ESCC cells

**Fig. 2:**
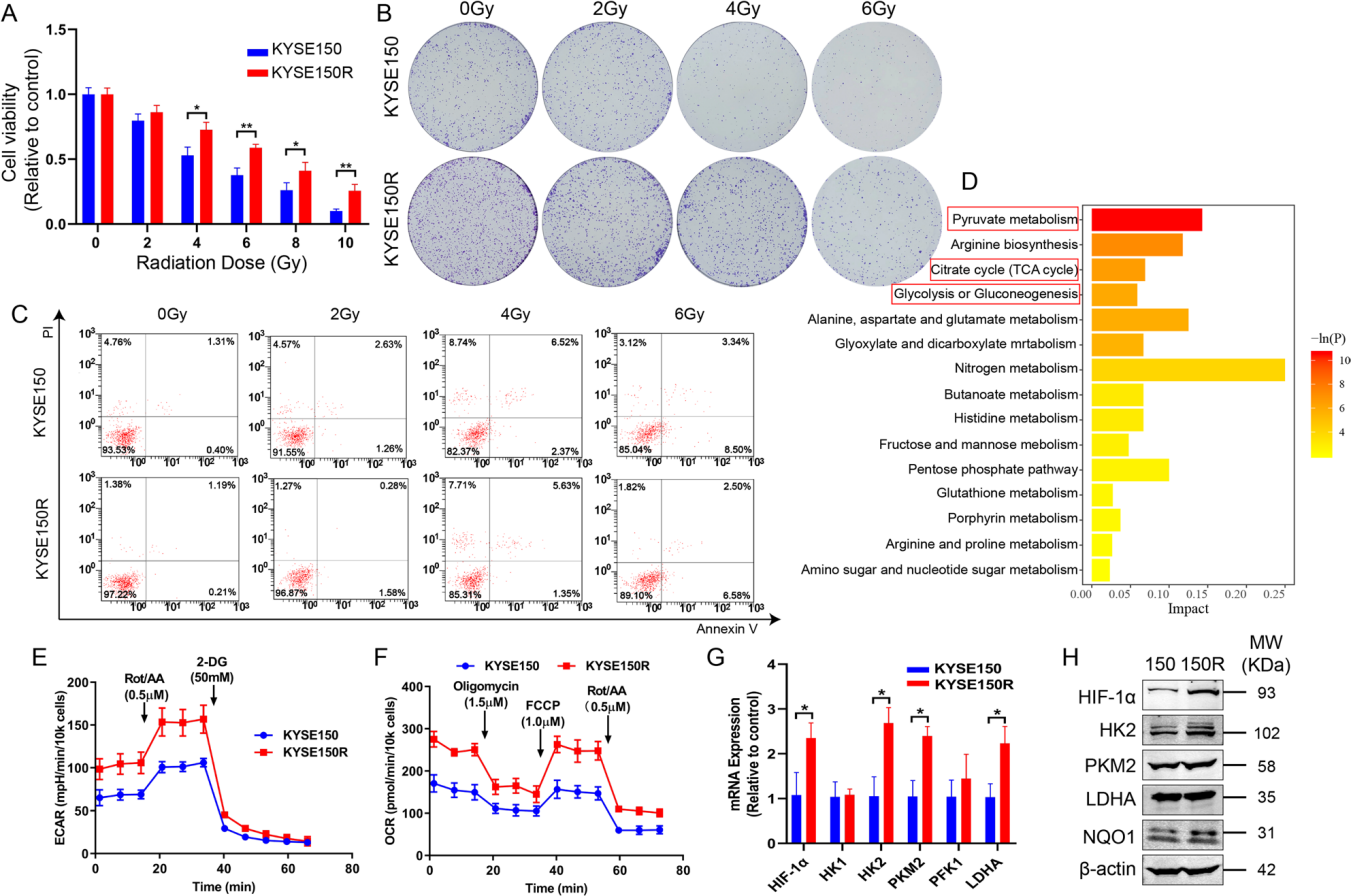
In vitro confirmation of the radioresistant phenotype and dual hypermetabolic state in ESCC cells. **A** Cell viability of KYSE150 and KYSE150R cells 48 h post-irradiation, assessed by MTS assay. **B** Clonogenic survival assay of KYSE150 and KYSE150R cells evaluated by crystal violet staining 10 days post-irradiation. **C** Apoptosis analysis via flow cytometry (Annexin V/PI staining) 48 h after exposure to varying radiation doses. **D** KEGG pathway enrichment analysis of differential metabolites between KYSE150 and KYSE150R cells. **E** Extracellular acidification rate (ECAR) measured via Seahorse XF96 analyzer to assess glycolytic capacity. **F** Oxygen consumption rate (OCR) measured to evaluate mitochondrial oxidative phosphorylation (OXPHOS). G Relative mRNA expression levels of HIF-1α, HK1, HK2, PKM2, and LDHA determined by RT-qPCR. **H** Protein expression levels of HIF-1α, HK2, PKM2, LDHA, and NQO1 detected by Western blotting. Data are mean ± SD from three independent experiments. * *P* < 0.05, ** P < 0.01, *** *P* < 0.001

We utilized KYSE150 and radioresistant KYSE150R cells to verify the clinical metabolic phenotype in vitro. Following irradiation, KYSE150R cells displayed increased viability, enhanced colony formation (Fig. 2A-B, Fig. S1A), and reduced apoptosis (Fig. 2C, Fig. S1B) compared to parental cells. Consistent with the patient data, targeted metabolomics of the cell lines revealed a significant enrichment in pyruvate metabolism, TCA cycle and glycolysis (Fig. 2D). This was functionally confirmed by real-time metabolic flux assays, which showed concurrent elevations in both extracellular acidification rate (ECAR) and oxygen consumption rate (OCR) in KYSE150R cells (Fig. 2E-F), indicating simultaneously hyperactive glycolysis and OXPHOS. Furthermore, mRNA and protein expression levels of key metabolic drivers (HIF-1α, HK2, PKM2, LDHA, and NQO1) were significantly upregulated in the resistant line (Fig. 2G-H, Fig. S1C-D). Thus, radioresistant ESCC cells adopt a coordinated dual hypermetabolic state to mitigate radiation-induced damage.

### Cepharanthine exhibits broad-spectrum and synergistic radiosensitization in ESCC

**Fig. 3:**
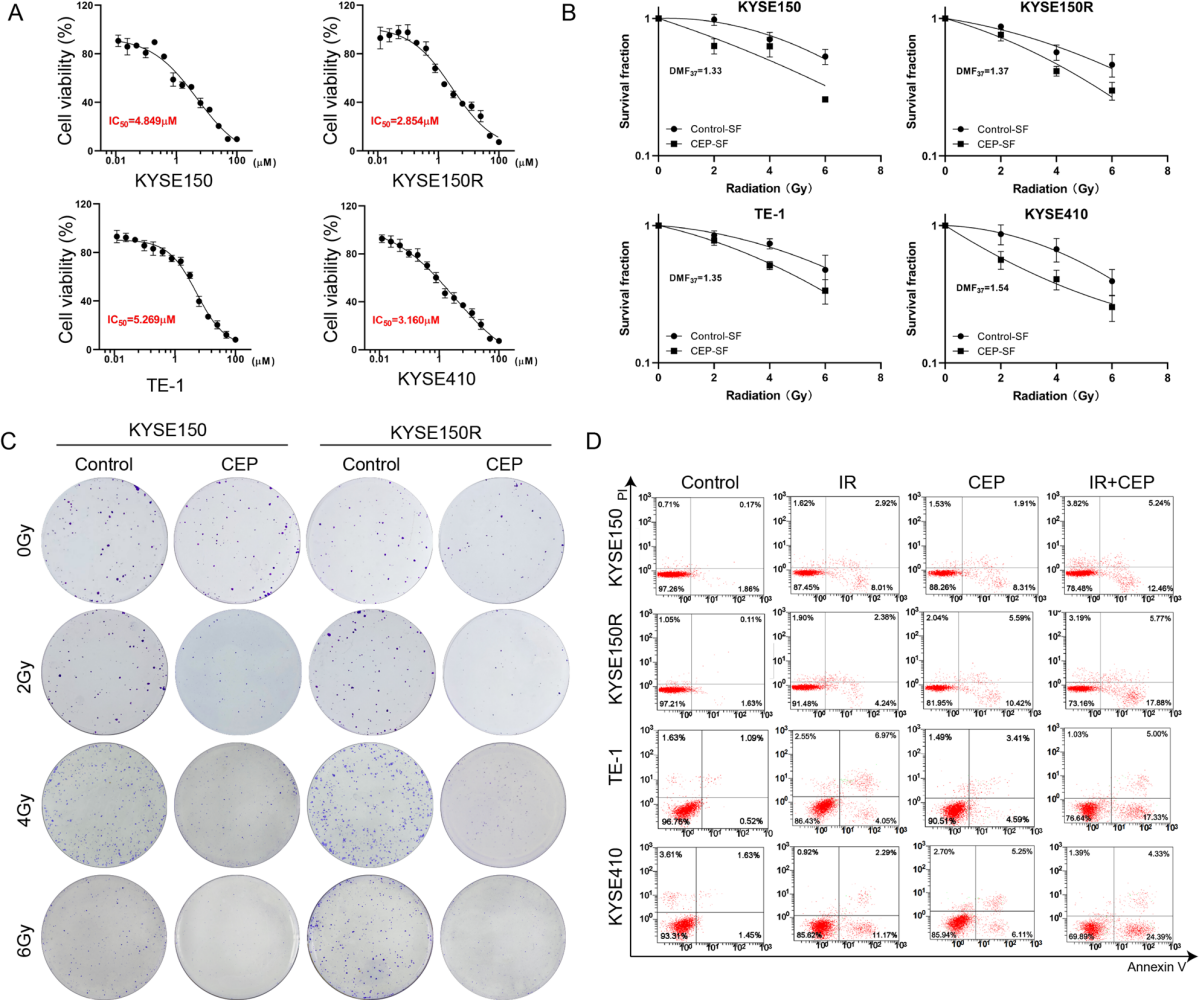
Cepharanthine (CEP) exerts broad-spectrum and synergistic radiosensitization across multiple ESCC cell lines. **A** Intrinsic cytotoxicity of CEP. Cell viability was measured via MTS assay in KYSE150, KYSE150R, TE-1, and KYSE410 cells to determine IC_50_ values and identify sub-lethal doses for combination treatments. **B-C** Clonogenic survival of ESCC cells exposed to escalating radiation doses (0, 2, 4, 6 Gy) with or without low-dose CEP treatment. (B) Survival curves fitted by the linear-quadratic (LQ) model for KYSE150, KYSE150R, TE-1, and KYSE410 cell lines. The dose-modifying factor at 37% survival (DMF_37_) is calculated from the respective LQ curves and displayed within each graph. A DMF_37_ > 1 indicates a synergistic radiosensitizing effect. (C) Representative clonogenic assay images for the KYSE150 and KYSE150R cell lines across the indicated radiation doses. **D** Flow cytometric analysis of apoptosis (Annexin V/PI staining) in KYSE150, KYSE150R, TE-1, and KYSE410 cell lines treated with vehicle, irradiation (IR), CEP, or their combination. Data are mean ± SD from three independent experiments.

We next evaluated the radiosensitizing potential of CEP, a natural bisbenzylisoquinoline alkaloid. Initial MTS assays across multiple ESCC cell lines (KYSE150, KYSE150R, TE-1, and KYSE410) revealed a dose-dependent cytotoxicity. Notably, the radioresistant KYSE150R line displayed a lower IC_50_ value compared to its parental counterpart, suggesting an inherent hypersensitivity to CEP (Fig. 3A).

To determine if CEP acts as a true radiosensitizer, we performed clonogenic survival assays using sub-lethal concentrations of CEP. Across all tested cell lines, the combination of CEP and escalating radiation doses significantly reduced macroscopic colony formation (Fig. 3C and Fig. S1E). When these survival fractions were mathematically fitted to the linear-quadratic (LQ) model, CEP treatment induced a downward shift in the survival curves across the entire cell line panel (Fig. 3B). The calculated dose-modifying factors at 37% survival (DMF_37_) were consistently greater than 1 across all tested cell lines, confirming a synergistic radiosensitizing effect rather than merely additive baseline toxicity (Fig. 3B). Consistent with the clonogenic data, flow cytometric analysis verified that the combination of CEP and irradiation induced a markedly higher rate of apoptosis compared to either monotherapy (Fig. 3D, Fig. S1F).

### CEP abrogates radioresistance by disrupting mitochondrial integrity and triggering oxidative lethal cascades

**Fig. 4:**
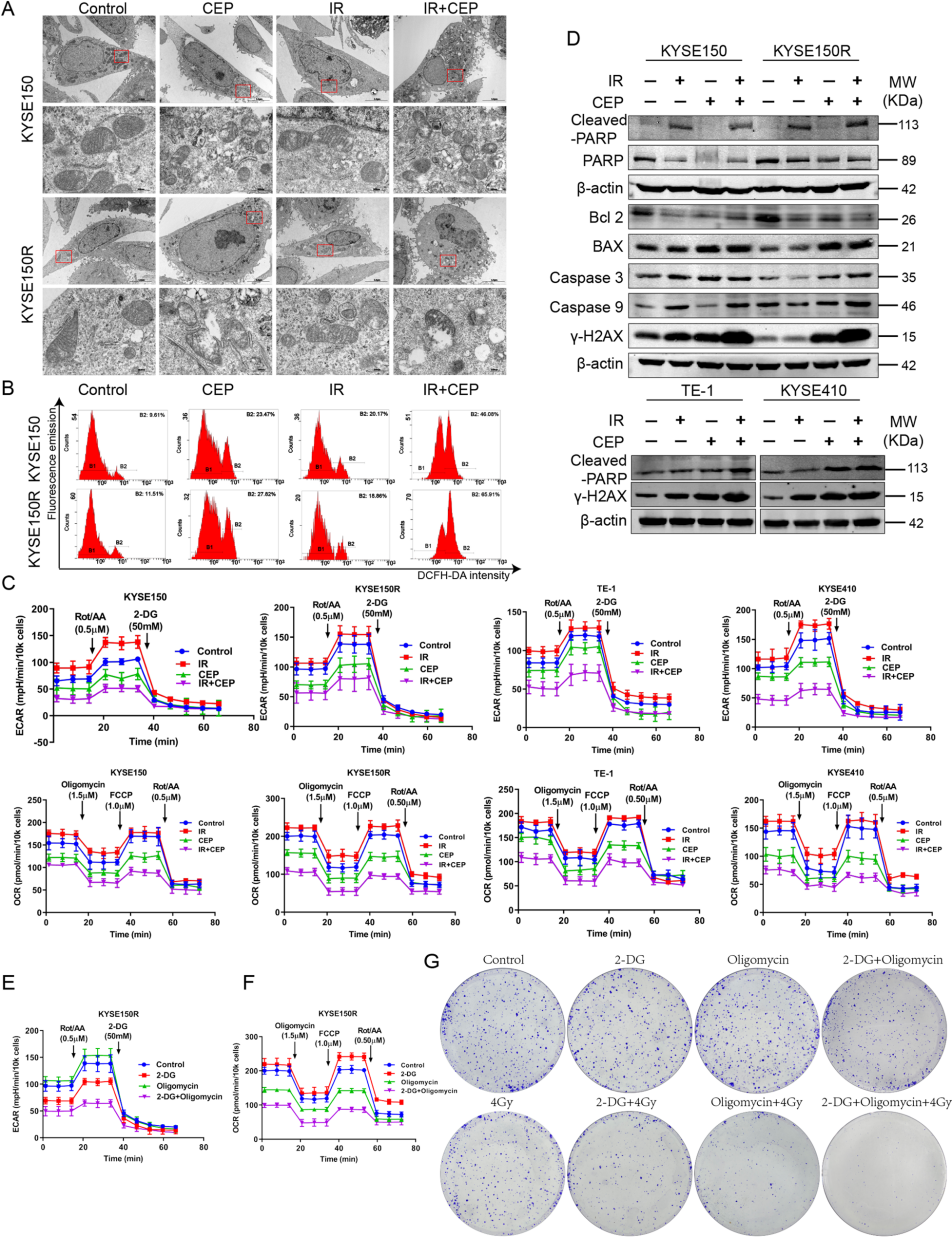
CEP sensitizes ESCC cells by disrupting mitochondrial integrity, collapsing energy metabolism, and driving oxidative DNA damage. **A** Representative TEM images showing mitochondrial ultrastructure in the paired KYSE150/150R model following the indicated treatments. Red boxes highlight fragmented cristae and severe vacuolization induced by CEP. **B** Flow cytometric analysis of intracellular ROS levels (DCFH-DA staining) in KYSE150 and KYSE150R cells across the four treatment groups (Control, CEP, IR, and IR + CEP). **C** Real-time metabolic flux analysis (ECAR and OCR) demonstrating the synergistic collapse of glycolysis and OXPHOS following combined treatment. **D** Western blot analysis evaluating DNA damage (γ-H2AX) and intrinsic apoptosis markers (Bcl-2, Bax, Caspase-9, Caspase-3, and PARP/cleaved PARP) across the ESCC cell line panel. β-actin served as the loading control. Data represent three independent experiments. **E-F** Real-time metabolic flux analysis (ECAR and OCR) validating pathway-specific inhibition in KYSE150R cells treated with 2-DG (1 mM), oligomycin (5 nM), or their combination. **G** Clonogenic survival assay of KYSE150R cells pre-treated with the indicated metabolic inhibitors prior to 4 Gy irradiation

To elucidate the subcellular mechanisms underlying CEP-mediated radiosensitization, we utilized our primary paired model (KYSE150 and KYSE150R) for high-resolution structural and reactive oxygen species (ROS) analyses. Transmission electron microscopy (TEM) revealed that CEP treatment caused severe physical disruption to mitochondrial ultrastructure, characterized by extensive cristae fragmentation and vacuolization (Fig. 4A). Consequent to this structural damage, the mitochondrial electron transport chain was impaired, leading to a significant intracellular accumulation of ROS. While radiation or CEP alone induced moderate ROS elevation, their combination triggered a marked oxidative burst (Fig. 4B, Fig. S1G). Notably, the downstream consequences of this severe mitochondrial damage were conserved across our broader panel of ESCC cell lines. Real-time metabolic flux analysis demonstrated that the CEP/irradiation combination markedly reduced the dual hypermetabolic phenotype, significantly suppressing both glycolytic capacity (ECAR) and mitochondrial respiration (OCR) in all tested cell lines, including the validation panel TE-1 and KYSE410 (Fig. 4C). Simultaneous measurement of OCR and ECAR further revealed that this combined treatment did not merely induce a compensatory metabolic shift (*e.g.*, upregulating glycolysis to rescue mitochondrial impairment); instead, it resulted in severe bioenergetic impairment and the depletion of cellular energy reserves in radioresistant cells. This metabolic arrest and oxidative stress ultimately initiated apoptotic cascades. Western blot analysis across the cell line panel confirmed that the combined treatment markedly upregulated the DNA double-strand break marker γ-H2AX and hyperactivated the intrinsic apoptotic pathway, evidenced by an increased Bax/Bcl-2 ratio and robust cleavage of Caspase-9, Caspase-3, and PARP (Fig. 4D, Fig. S1H-K).

To assess the functional contribution of this metabolic duality to the radioresistant phenotype, KYSE150R cells were subjected to targeted metabolic blockade using 2-DG (a glycolysis inhibitor, 1 mM) and oligomycin (an OXPHOS inhibitor, 5 nM). Real-time metabolic flux analysis confirmed pathway-specific inhibition, demonstrating that the combination of 2-DG and oligomycin simultaneously suppressed both ECAR and OCR (Fig. 4E-F). We next evaluated the impact of this targeted intervention on radiosensitivity. Clonogenic survival assays showed that individual or combined metabolic inhibitors exerted minimal baseline cellular toxicity in the absence of radiation. However, combining either 2-DG or oligomycin with 4 Gy irradiation decreased colony formation relative to irradiation alone. The concurrent blockade of both glycolysis and OXPHOS prior to irradiation resulted in a synergistic reduction in clonogenic survival, yielding the lowest colony formation rates among all groups (Fig. 4G, Fig. S1L). These data indicate that the simultaneous disruption of dual energetic pathways is a fundamental requirement to reverse the radioresistant phenotype in ESCC.

### Multi-omics integration identifies p70S6K as the direct target of CEP and a prognostic biomarker for radioresistance

**Fig. 5:**
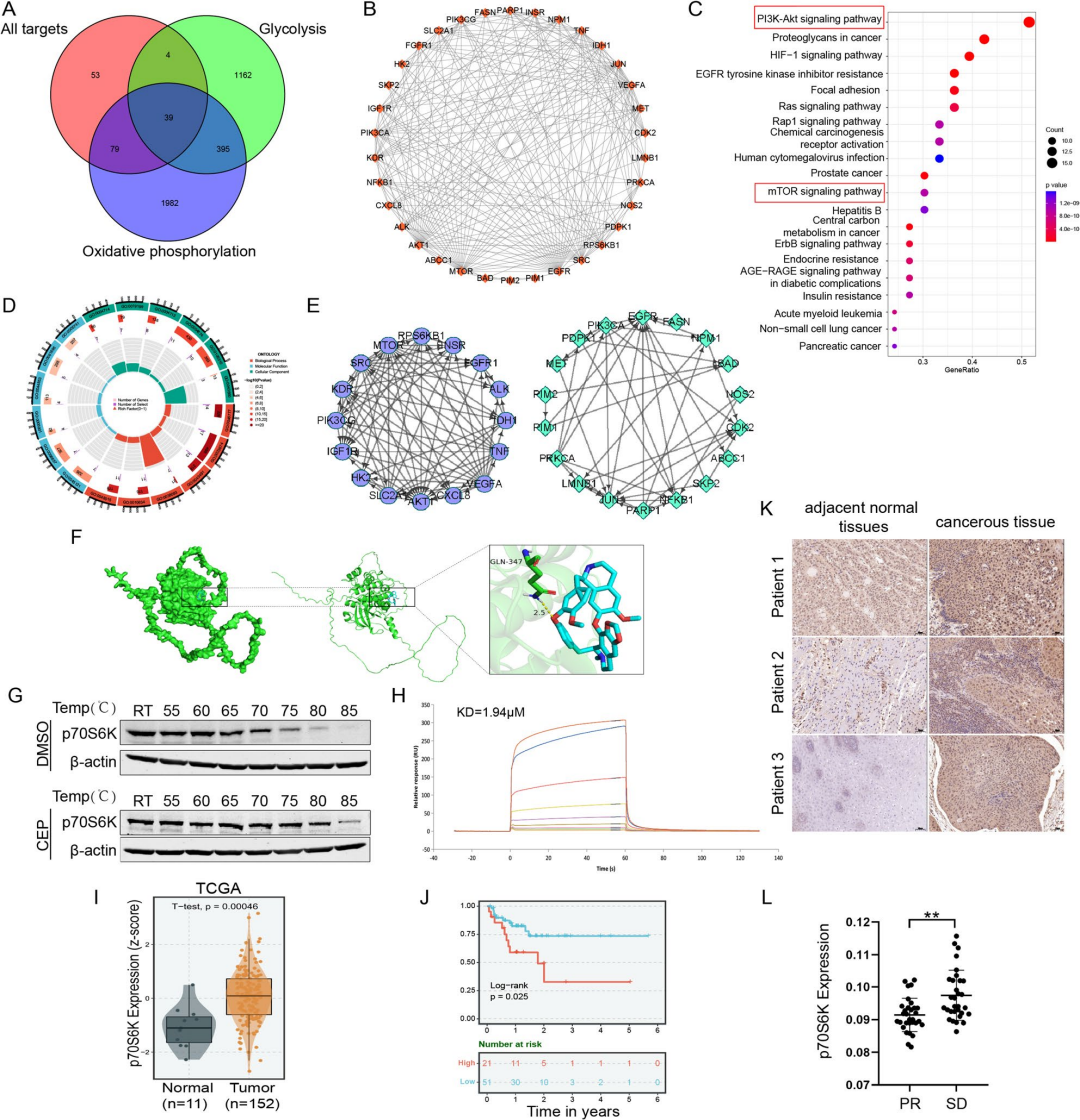
CEP directly targets p70S6K, a master metabolic regulator and prognostic biomarker in ESCC. **A** Venn diagram illustrating the intersection of predicted CEP targets with glycolysis and oxidative phosphorylation (OXPHOS) gene sets. **B** Protein-protein interaction (PPI) network of the 39 overlapping core genes. **C** KEGG pathway enrichment analysis of the intersecting genes, prominently highlighting the PI3K/Akt and mTOR signaling pathways. **D** Circular plot showing Gene Ontology (GO) enrichment analysis of the overlapping targets. **E** Key sub-network clusters extracted from the overarching PPI network utilizing the MCODE algorithm. **F** Molecular docking model simulating the binding interface and affinity between CEP and the p70S6K kinase domain (interacting at the Gln347/Q347 residue). **G** Cellular thermal shift assay (CETSA) demonstrating that CEP binding enhances the thermal stability of p70S6K in intact cells. **H** Surface plasmon resonance (SPR) sensorgram confirming the direct binding affinity (*K*_*D*_ = 1.94 µM) between CEP and purified p70S6K protein. **I** Analysis of TCGA data showing elevated p70S6K mRNA expression in ESCC tumor tissues (*n* = 152) compared to normal esophageal tissues (*n* = 11). **J** Kaplan-Meier curves for disease-free survival (DFS) in ESCC patients stratified by high (*n* = 21) versus low (*n* = 51) p70S6K expression levels. **K** Representative immunohistochemistry (IHC) images comparing p70S6K protein expression in paired cancerous and adjacent normal tissues (Scale bars, 50 μm). **L** Quantification of p70S6K levels in radiosensitive (PR, *n* = 31) versus radioresistant (SD, *n* = 31) ESCC patients

Having established that CEP functionally disrupts the dual hypermetabolic state, we employed a multi-omics approach to trace the upstream molecular target. Comprehensive metabolomic profiling showed that CEP treatment induced global perturbations across both glycolytic and mitochondrial metabolic pathways in KYSE150 and KYSE150R cells (Fig. S2A-B). Concurrently, transcriptomic profiling coupled with Gene Set Enrichment Analysis (GSEA) revealed a suppression of mitochondrial gene expression and translation networks following CEP intervention (Fig. S2C-D). To identify the upstream signaling node responsible for driving these alterations, we conducted a network pharmacology analysis. Intersecting CEP’s predicted pharmacological targets with glycolysis and OXPHOS core gene sets identified 39 overlapping targets (Fig. 5A-B). KEGG pathway enrichment of these genes highlighted the PI3K/Akt and mTOR signaling cascades as the central regulatory hubs (Fig. 5C-E, Fig. S2E-H). Given p70S6K’s role as a metabolic effector within this axis, we investigated its physical interaction with CEP. Molecular docking predicted an interaction at the GLN347 (Q347) residue of the p70S6K kinase domain (binding energy: -8.3 kcal/mol; Fig. 5F). We validated this direct engagement experimentally: CEP treatment enhanced the thermal stability of p70S6K in intact cells (CETSA, Fig. 5G), and surface plasmon resonance (SPR) quantified a direct binding affinity with a *K*_*D*_ of 1.94 µM (Fig. 5H).

We then evaluated the clinical significance of p70S6K in ESCC. Analysis of the TCGA database revealed an upregulation of p70S6K mRNA in tumor tissues compared to normal mucosa (Fig. 5I), which was corroborated at the protein level by immunohistochemistry in our patient cohort (Fig. 5K). Clinically, high p70S6K expression was associated with poorer disease-free survival (Fig. 5J). Furthermore, circulating p70S6K levels were elevated in radioresistant (SD) patients compared to radiosensitive (PR) responders (Fig. 5L). These data identify p70S6K as a therapeutic target of CEP and a clinical biomarker for ESCC radioresistance.

### CEP inhibits the PI3K/Akt/mTOR signaling axis and triggers ubiquitin-proteasomal degradation of p70S6K

**Fig. 6:**
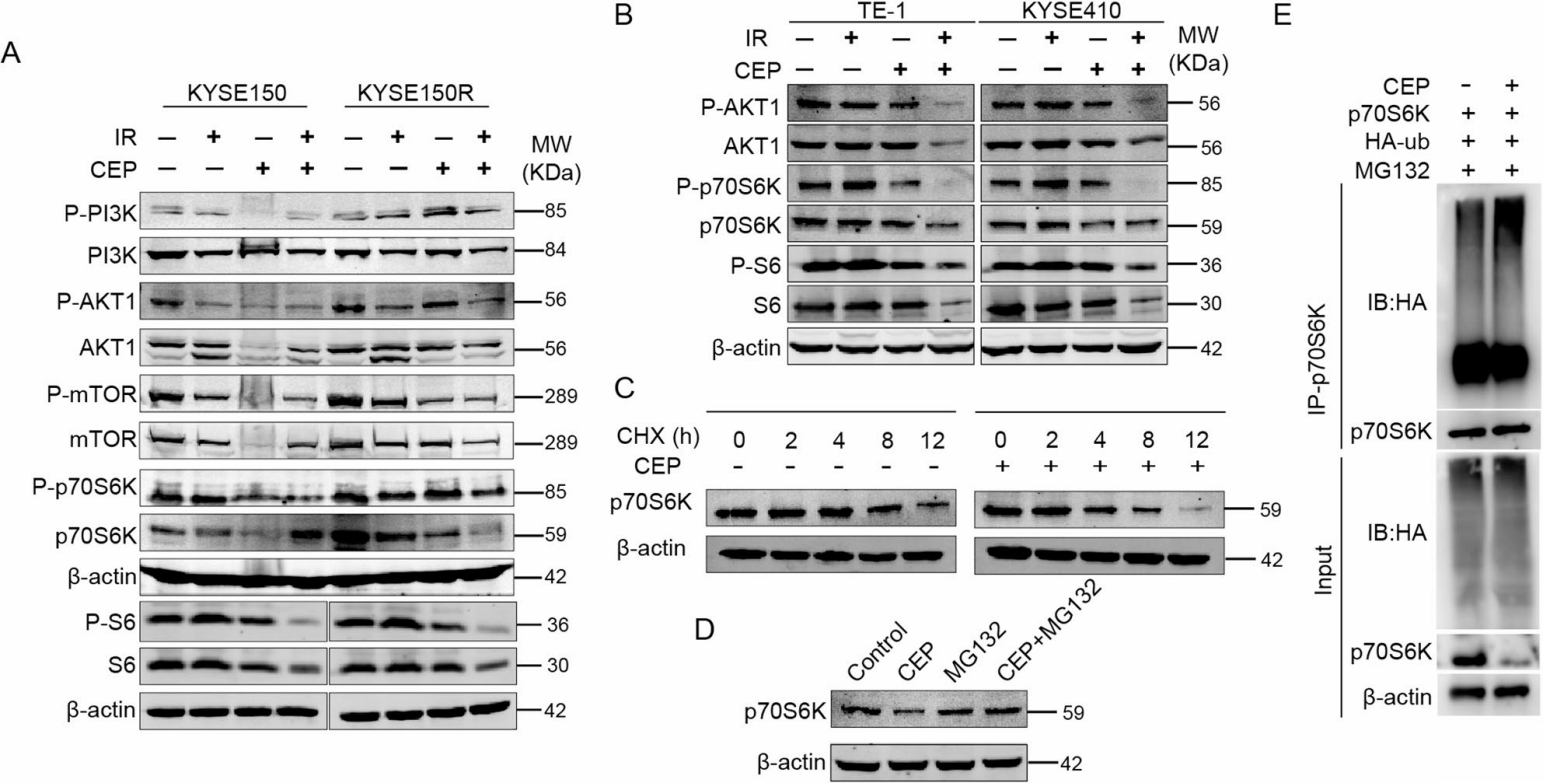
CEP inhibits the PI3K/Akt/mTOR pathway and promotes the ubiquitin-proteasomal degradation of p70S6K. **A-B** Western blot analysis of total and phosphorylated PI3K, AKT1, mTOR, p70S6K, and S6 in KYSE150, KYSE150R (A), TE-1, and KYSE410 (**B**) cells. Cells were treated with vehicle or CEP, in the presence or absence of irradiation (IR). β-actin served as the loading control. **C** Cycloheximide (CHX) chase assay evaluating p70S6K protein stability. Cells were treated with CHX in the presence or absence of CEP for the indicated times (0, 2, 4, 8, and 12 h) prior to immunoblotting. **D** Western blot analysis evaluating the rescue effect of proteasome inhibition. Cells were pre-treated with the proteasome inhibitor MG132 followed by CEP treatment. **E** Cell-based ubiquitination assay demonstrating CEP-induced polyubiquitination of p70S6K. Cells co-transfected with p70S6K and HA-tagged ubiquitin (HA-Ub) plasmids were treated with CEP or vehicle in the presence of MG132. Cell lysates were immunoprecipitated (IP) with an anti-p70S6K antibody and immunoblotted (IB) with an anti-HA antibody. Input lysates were analyzed for baseline protein expression

To elucidate the downstream molecular consequences of CEP treatment, we evaluated the PI3K/Akt/mTOR signaling cascade across four ESCC cell lines (KYSE150, KYSE150R, TE-1, and KYSE410). Western blot analysis indicated that acute CEP exposure, both alone and in combination with irradiation, suppressed the phosphorylation of PI3K, AKT1, mTOR, p70S6K, and its downstream effector S6 (Fig. 6A-B, Fig. S3A-F).

In addition, CEP treatment also resulted in a reduction of total p70S6K protein levels. To determine whether this reduction was due to altered protein stability, we performed a cycloheximide (CHX) chase assay. The results showed that CEP accelerated the degradation rate of p70S6K, shortening its protein half-life (Fig. 6C, Fig. S3G). Co-treatment with the proteasome inhibitor MG132 reversed this CEP-induced downregulation, indicating a proteasome-dependent clearance mechanism (Fig. 6D, Fig. S3H). Furthermore, a cell-based ubiquitination assay confirmed that CEP treatment enhanced the accumulation of polyubiquitinated p70S6K species compared to the vehicle control (Fig. 6E). These biochemical data demonstrate that CEP not only inhibits the kinase cascade within the PI3K/Akt/mTOR axis but also triggers the ubiquitin-proteasomal degradation of its direct target, p70S6K.

### p70S6K is the functional executor mediating CEP-induced metabolic suppression and radiosensitization

**Fig. 7:**
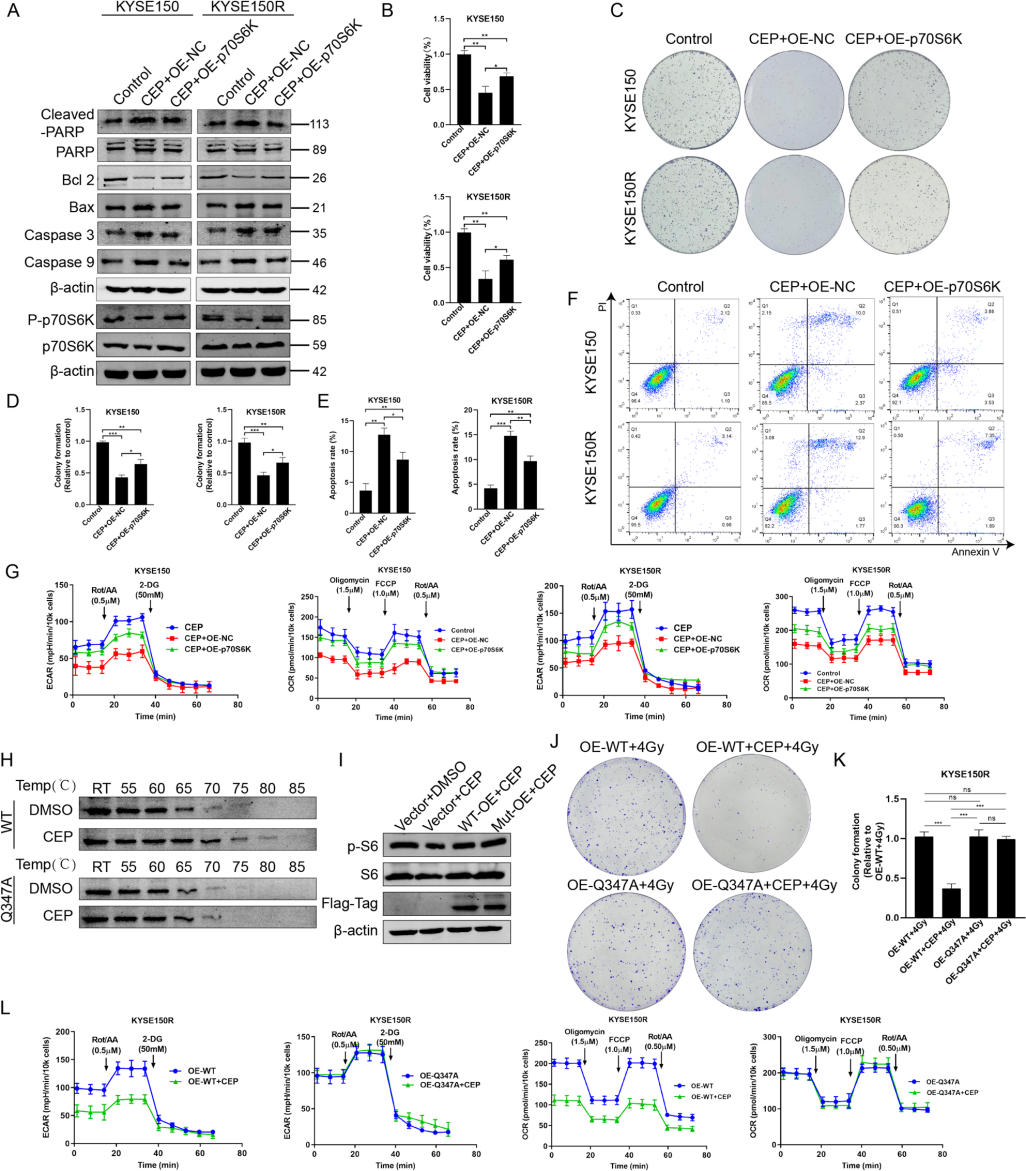
p70S6K mediates CEP-induced metabolic suppression, depending on the Q347 binding pocket. **A** Western blot analysis of p70S6K, Phospho-p70S6K, and downstream apoptosis-related proteins in OE-NC and OE-p70S6K cells following CEP exposure. β-actin served as the loading control. **B** Cell viability of KYSE150 and KYSE150R cells transfected with empty vector (OE-NC) or wild-type p70S6K (OE-p70S6K), followed by treatment with vehicle or CEP. **C-D** Representative images (C) and quantification (D) of colony formation assays in the indicated cell groups. **E-F** Apoptosis rates quantified by bar charts (E) and representative flow cytometry plots (F) of cells treated with or without CEP under p70S6K overexpression. **G** Real-time metabolic flux analysis measuring the ECAR and OCR in OE-NC and OE-p70S6K cells. **H** CETSA evaluating the thermal stability of WT and Q347A mutant p70S6K proteins in the presence of vehicle or CEP across a temperature gradient. **I** Immunoblot analysis of downstream S6 phosphorylation (p-S6) and total S6 in KYSE150R cells transfected with empty vector, wild-type p70S6K (WT-OE), or Q347A mutant p70S6K (Mut-OE) plasmids, followed by DMSO or CEP treatment. Flag-tag confirms the expression of the exogenous p70S6K constructs, and β-actin serves as the loading control. **J-K** Representative images (**J**) and relative quantification (K) of clonogenic survival. OE-WT and OE-Q347A cells were treated with vehicle or CEP prior to a single fixed dose of irradiation (4 Gy). Data are normalized to the OE-WT+4 Gy group. **L** ECAR and OCR profiles of KYSE150R cells expressing wild-type p70S6K (OE-WT) or the Q347A mutant (OE-Q347A), followed by treatment with vehicle or CEP. All quantitative data are presented as mean ± SD from three independent experiments. * *P* < 0.05, ** *P* < 0.01, *** *P* < 0.001; ns, not significant

To determine whether p70S6K is the functional target of CEP, we performed genetic rescue experiments in ESCC cells. Western blot analysis verified the restoration of p70S6K and Phospho-p70S6K protein expression, alongside the reversal of downstream apoptotic markers, in CEP-treated cells following lentiviral overexpression of wild-type p70S6K (OE-p70S6K) (Fig. 7A, Fig. S3I-M). Phenotypically, p70S6K overexpression mitigated CEP-induced cell viability inhibition (Fig. 7B), restored colony formation (Fig. 7C-D), and reduced apoptosis rates (Fig. 7E-F). Furthermore, metabolic flux analysis showed that OE-p70S6K cells resisted the CEP-induced suppression of ECAR and OCR, maintaining functional bioenergetics (Fig. 7G).

To define the structural requirement of the predicted binding pocket for these effects, we generated a p70S6K-Q347A mutant and assessed its interaction with CEP. Cellular thermal shift assays (CETSA) showed that CEP enhanced the thermal stability of WT p70S6K but failed to confer thermal protection to the Q347A mutant, indicating a loss of direct physical engagement (Fig. 7H). In agreement with this physical uncoupling, immunoblot analysis using Flag-tagged constructs revealed that CEP suppressed downstream S6 phosphorylation in vector-transfected cells, whereas cells expressing the Q347A mutant maintained p-S6 signaling during CEP treatment (Fig. 7I, Fig. S3N).

Functionally, this loss of target engagement rendered the cells refractory to CEP. Real-time bioenergetic analysis indicated that the Q347A mutation prevented CEP-induced metabolic suppression, with cells sustaining baseline ECAR and OCR levels (Fig. 7L). Furthermore, in clonogenic survival assays following a single fixed dose of irradiation (4 Gy), the Q347A mutation negated the radiosensitizing effect of CEP, resulting in colony formation rates comparable to the untreated controls (Fig. 7J-K). Collectively, these data demonstrate that the Q347 residue is structurally required for CEP to inhibit the p70S6K-driven metabolic network and reverse radioresistance.

### CEP synergizes with radiotherapy in vivo across immunodeficient and immunocompetent models

**Fig. 8:**
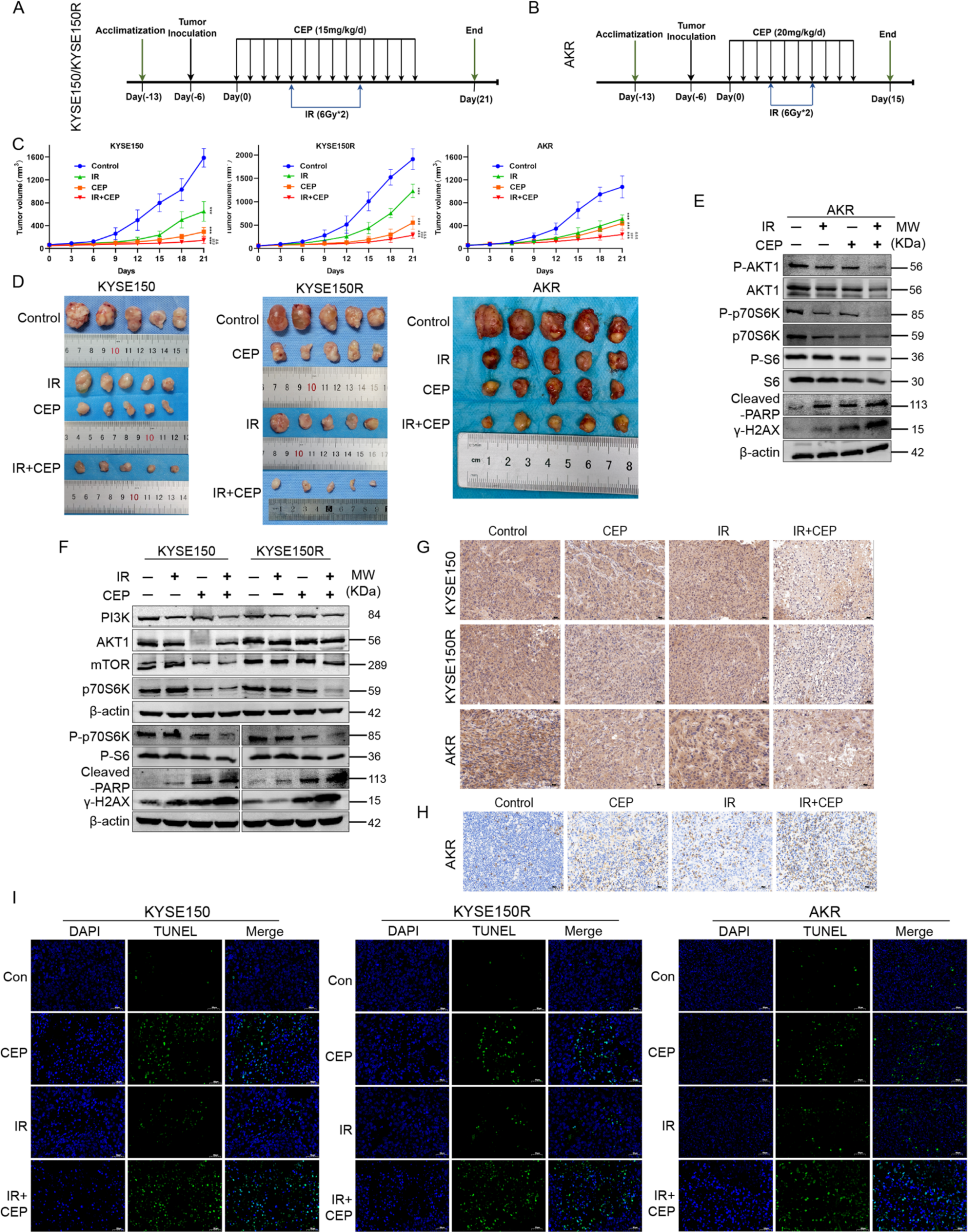
CEP exerts synergistic radiosensitization and suppresses the p70S6K axis in vivo. **A-B** Schematic timelines of the treatment protocols for the immunodeficient CDX models (KYSE150 and KYSE150R) (A) and the immunocompetent syngeneic model (AKR) (B). The schematic diagrams were created using FigDraw. **C** Tumor growth curves for the KYSE150, KYSE150R, and AKR models across the designated treatment groups. **D** Representative macroscopic images of excised tumors at the experimental endpoints. **E-F** Western blot analysis of the PI3K/Akt/mTOR/p70S6K pathway and downstream apoptosis/DNA damage markers in tumor tissues from the indicated models. β-actin served as the loading control. **G** Representative IHC staining of p70S6K in tumor sections across the three models. Scale bars: 50 μm. **H** IHC staining evaluating CD8^+^ T cell infiltration in the AKR syngeneic tumor tissues. Scale bars: 50 μm. **I** Representative TUNEL fluorescence staining evaluating cellular apoptosis. Blue: DAPI (nuclei); Green: TUNEL-positive apoptotic cells. Scale bars: 50 μm. Data are presented as mean ± SD. *** *P* < 0.001 compared with the Control group; ### *P* < 0.001 compared with the IR group; &&& *P* < 0.001 compared with the CEP group

To evaluate the translational potential of CEP as a radiosensitizer, we utilized human cell-derived xenograft (CDX) models (KYSE150 and KYSE150R) and a syngeneic immunocompetent model (AKR). Following the establishment of palpable tumors, mice were treated with vehicle, CEP (dose-optimized for each model), fractionated local irradiation (6 Gy × 2 fractions), or the combination therapy (Fig. 8A-B). Across all three models, the concurrent administration of CEP and irradiation significantly suppressed tumor growth kinetics and reduced terminal tumor volumes more effectively than either monotherapy alone (Fig. 8C-D).

We next assessed the molecular and histological alterations within the tumor tissues. Western blot analysis of in vivo tumor lysates demonstrated that CEP treatment effectively inhibited key components of the PI3K/Akt/mTOR/p70S6K signaling cascade and upregulated markers of DNA damage (γ-H2AX) and apoptosis (Cleaved PARP), consistent with our in vitro findings (Fig. 8E-F, Fig. S4C-H). This target suppression was further corroborated by immunohistochemical (IHC) analysis, which showed a marked decrease in in situ p70S6K expression within the tumor sections (Fig. 8G).

Morphologically, the combination treatment led to disrupted tissue architecture (Fig. S4A) and a notable reduction in the proliferation marker Ki-67 (Fig. S4B). Furthermore, in the immunocompetent AKR model, IHC analysis revealed a markedly increased infiltration of CD8^+^ T cells within the tumor microenvironment of the combination group (Fig. 8H), suggesting that this intervention also promotes a local immune-activating effect. These molecular and microenvironmental alterations collectively culminated in a widespread induction of apoptosis, as evidenced by extensive TUNEL-positive staining (Fig. 8I).

In addition, histological evaluation (H&E staining) of vital organs, including the heart, liver, spleen, lungs, and kidneys, revealed no discernible morphological lesions or pathological abnormalities across any of the treatment groups (Fig. S4I-K). This confirms that the therapeutic regimens were well-tolerated without inducing overt systemic toxicity. Collectively, these robust data demonstrate the in vivo efficacy and safety of CEP in reversing ESCC radioresistance.

## Discussion

The acquisition of radioresistance in ESCC dictates treatment failure and poor clinical outcomes [3]. While previous studies have reported the phenomenological radiosensitizing properties of Cepharanthine (CEP) in various malignancies—primarily attributing its effects to broad mechanisms such as ROS induction or cell cycle arrest [11, 12, 17, 18]—the present study provides new mechanistic insights. Our study delineates a precise structural, metabolic, and immunological axis. Specifically, we define a novel “metabolic duality” that drives ESCC radioresistance [8–10] and demonstrate that CEP directly targets the Q347 residue of p70S6K, triggering its ubiquitin-proteasomal degradation. This targeted clearance induces severe bioenergetic impairment and remodels the tumor immune microenvironment, providing a robust mechanistic rationale for evaluating CEP as a multifaceted radiosensitizer.

Our clinical multi-omics data challenge the classical Warburg-centric dogma in therapy resistance. While aerobic glycolysis remains a hallmark of naive tumors, we observed that radioresistant ESCC patients and established cell lines adopt a coordinated hyperactivation of both glycolytic flux and oxidative phosphorylation (OXPHOS) to withstand treatment pressure [19–21]. This “metabolic duality” operates as a sophisticated survival network [22]: Glycolytic flux supplies immediate ATP and macromolecular precursors, while enhanced OXPHOS meets the high energetic demands of radiation-induced DNA repair [23]. These findings align with growing evidence across malignancies showing that therapy-resistant subpopulations often display heightened mitochondrial dependence [24, 25]. Pharmacological intervention with CEP effectively disrupted this dual dependency [26–28]. Unlike traditional antimetabolites that target individual enzymes, the direct binding of CEP to p70S6K abrogates the dual metabolic dependencies of radioresistant cells through the concurrent attenuation of both major bioenergetic pathways.

Furthermore, our mechanistic interrogation elevates CEP from a generic natural product to a targeted protein degrader. Through SPR, CETSA, and site-directed mutagenesis (Q347A), we confirmed that CEP does not merely inhibit kinase activity, but physically engages the p70S6K kinase domain to initiate ubiquitin-dependent degradation. Recent advances in precision oncology have highlighted that targeted protein degradation (TPD) via the ubiquitin-proteasome system offers therapeutic advantages over traditional occupancy-based inhibitors, effectively circumventing compensatory kinase activation and therapeutic resistance [29]. Interestingly, while acute in vitro CEP treatment primarily suppresses the phosphorylation of upstream PI3K and AKT1, prolonged in vivo exposure resulted in a concomitant reduction of their total protein levels. The PI3K/Akt/mTOR/p70S6K axis operates as a highly integrated macromolecular scaffold rather than a simple linear cascade [30, 31]. We hypothesize that the rapid and continuous CEP-induced degradation of p70S6K—the central metabolic effector—disrupts essential structural feedback loops. Over time, this chronic target depletion potentially triggers the destabilization and ultimate collapse of the broader upstream signaling complex, thereby accelerating radiosensitization [32, 33]. Resolving the exact architecture of this feedback network through structural proteomics remains an imperative direction for future research.

The translational significance of our findings is strongly reinforced by our in vivo models. While immunodeficient xenografts validated the core metabolic mechanism, our syngeneic AKR/C57BL/6 model provided critical insights into the tumor microenvironment. Furthermore, the preliminary observation of increased CD8^+^ T cell infiltration following the combined intervention suggests favorable modulation of the immune microenvironment. This secondary effect warrants future independent investigation, as it aligns consistently with the established paradigm of radiation-induced immunogenic cell death (ICD) [34].

Despite these promising findings, several limitations of the present study must be acknowledged. First, while our in vivo models effectively validated the core metabolic and tumor-suppressive mechanisms, the inherent biological barriers frequently encountered in establishing patient-derived xenografts (PDXs) from heavily pretreated, refractory ESCC specimens constrained our ability to evaluate broader inter-patient heterogeneity. Furthermore, while circulating p70S6K emerged as a robust prognostic biomarker in our cohort [35–38], its predictive validity necessitates rigorous testing in large-scale, prospective multicenter trials. Finally, high-resolution X-ray crystallography or cryo-EM structures are required to map the spatial coordinates of the CEP-p70S6K complex to guide future rational drug optimization.

In conclusion, our study identifies “metabolic duality” as a fundamental driver of radioresistance in ESCC. We demonstrate that CEP disrupts this dual bioenergetic dependency by physically engaging and inducing the targeted degradation of p70S6K. By elucidating this precise structural and metabolic mechanism, we provide a strong pharmacological rationale for repurposing CEP as a potent radiosensitizer. Given its established clinical safety profile, CEP represents a viable and actionable therapeutic strategy to overcome radioresistance in advanced ESCC.

## Supplementary Information

Below is the link to the electronic supplementary material.

Supplementary Material 1

Supplementary Material 2

## Data Availability

Data will be made available on reasonable request.

## Abbreviations

ESCC: esophageal squamous cell carcinoma
OXPHOS: oxidative phosphorylation
ROS: reactive oxygen species
CEP: cepharanthine
ELISA: enzyme-linked immunosorbent assay
IHC: immunohistochemistry
FBS: fetal bovine serum
RT‒qPCR: real-time quantitative polymerase chain reaction
IR: ionizing radiation
HRP: horseradish peroxidase
TMB: tetramethylbenzidine
H&E: hematoxylin and eosin
ECAR: extracellular acidification rate
OCR: oxygen consumption rate
ANOVA: one-way analysis of variance
KEGG: Kyoto Encyclopedia of Genes and Genomes
PR: partial response
SD: stable disease
SMPDB: small-molecule pathway database
PI: propidium iodide
DEGs: differentially expressed genes
GSEA: gene set enrichment analysis
GO: Gene Ontology
TEM: transmission electron microscopy
BP: biological process
CC: cellular component
MF: molecular function
PPI: protein‒protein interaction
p70S6K: ribosome S6 protein kinase
PFS: progression-free survival
DFS: disease-free survival
CETSA: cellular thermal shift assay
SPR: surface plasmon resonance
CDX: human cell-derived xenograft
2-DG: 2-Deoxy-D-glucose
DMF37: dose-modifying factor at 37% survival
CHX: cycloheximide
