# Supplementary material for: Repurposing cepharanthine as a radiosensitizer in esophageal squamous cell carcinoma through dual metabolic intervention and direct targeting of p70s6K
Source: J Transl Med. 2026 Jul 3;24:893. doi: 10.1186/s12967-026-08550-y (PMC13359829; doi:10.1186/s12967-026-08550-y)
Supplement: Supplementary file 1 — Supplementary Material 1 [file 12967_2026_8550_MOESM1_ESM.doc]

**Additional Files**

**Supplementary Figures**


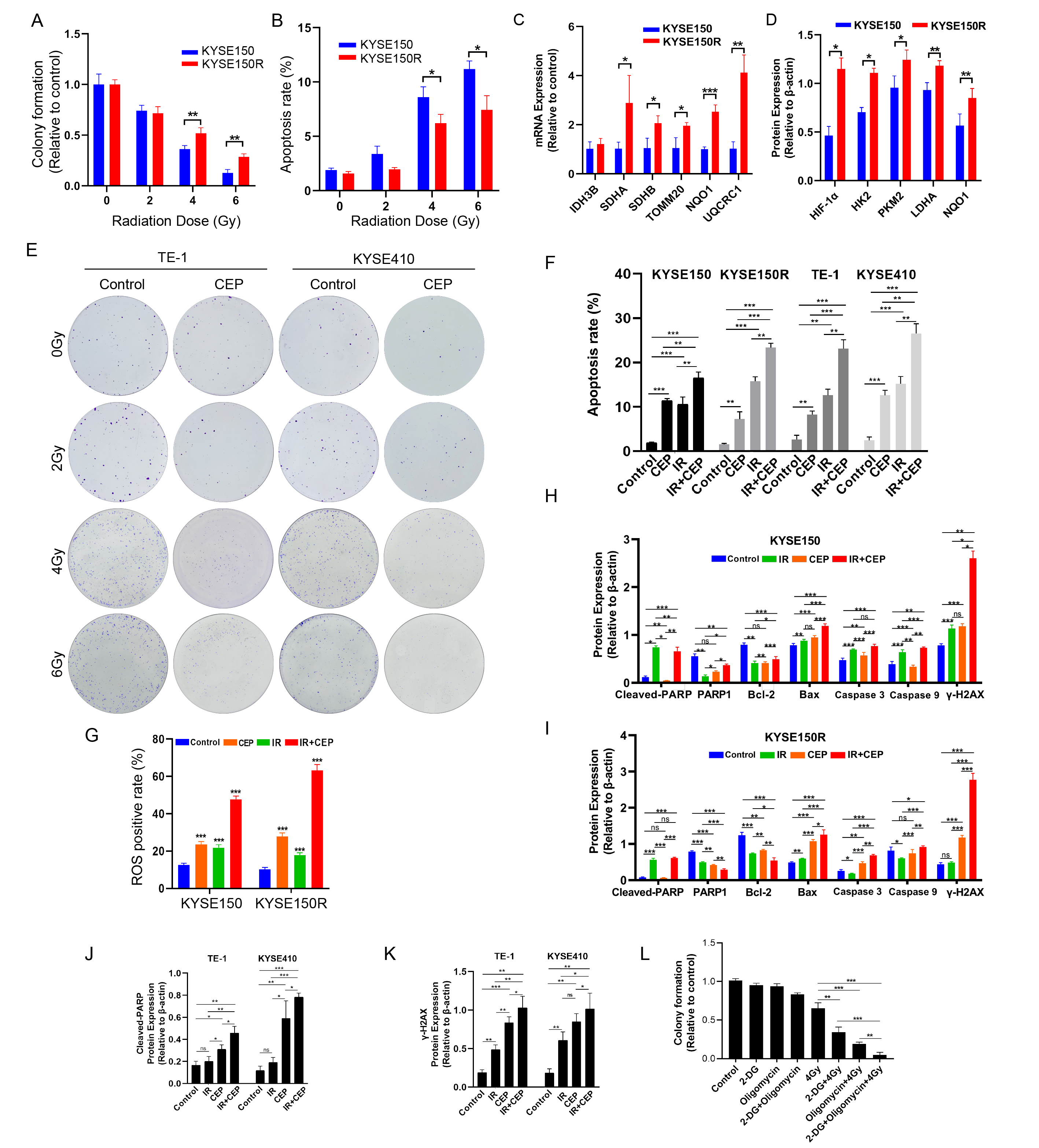


Figure S1. A-B Quantitative analysis of clonogenic survival and apoptosis rate in KYSE150 and KYSE150R cells. C Quantitative RT-qPCR analysis of genes related to glycolysis and mitochondrial metabolism in KYSE150 and KYSE150R cells. D Quantitative western blotting analysis of HIF-1α, HK2, PKM2, LDHA and NQO1 protein expression levels in KYSE150 and KYSE150R cells. E Representative clonogenic assay images for the KYSE150 and KYSE150R cell lines across the indicated radiation doses. F Flow cytometric analysis of apoptosis in KYSE150, KYSE150R, TE-1, and KYSE410 cell lines following the indicated treatments. G Flow cytometry analysis of intracellular ROS levels in KYSE150 and KYSE150R cells following the indicated treatments. H-K Quantitative western blotting analysis of γ-H2AX and intrinsic apoptosis markers (Bax, Bcl-2, cleaved Caspase-9/-3, and PARP) across the ESCC cell line panel. L Representative clonogenic assay images of KYSE150R cells following targeted metabolic blockade (2-DG and/or oligomycin) with or without 4 Gy irradiation. * *P* < 0.05, ** *P* < 0.01, *** *P* < 0.001.


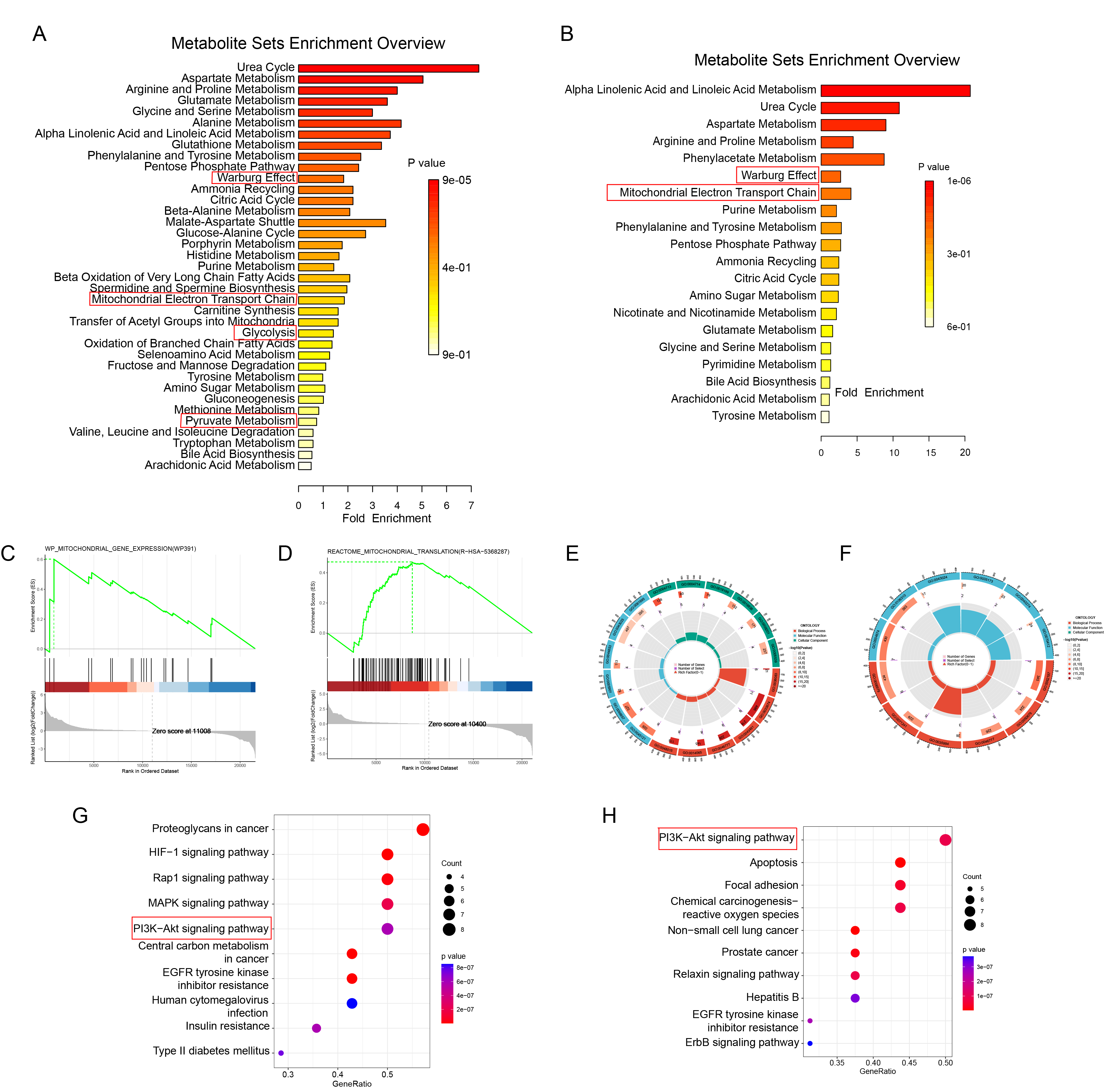


Figure S2. Multi-omics profiling and network pharmacology analysis of CEP-induced alterations.

A-B Metabolite set enrichment analysis highlighting significant perturbations in glycolytic pathways (e.g., Warburg Effect, Pyruvate Metabolism) and mitochondrial function (e.g., Mitochondrial Electron Transport Chain) following CEP treatment in KYSE150 and KYSE150R cells. C-D Gene Set Enrichment Analysis (GSEA) plots demonstrating the suppression of mitochondrial gene expression (C) and mitochondrial translation (D) signatures induced by CEP intervention.

E-F Circular plots illustrating the significantly enriched Gene Ontology (GO) terms—categorized by biological processes (BP), molecular functions (MF), and cellular components (CC)—for the identified overlapping target clusters (Cluster 1 and Cluster 2). G-H Bubble plots of KEGG pathway enrichment analysis revealing the PI3K/Akt signaling pathway as a highly enriched and central regulatory hub among the predicted targets..


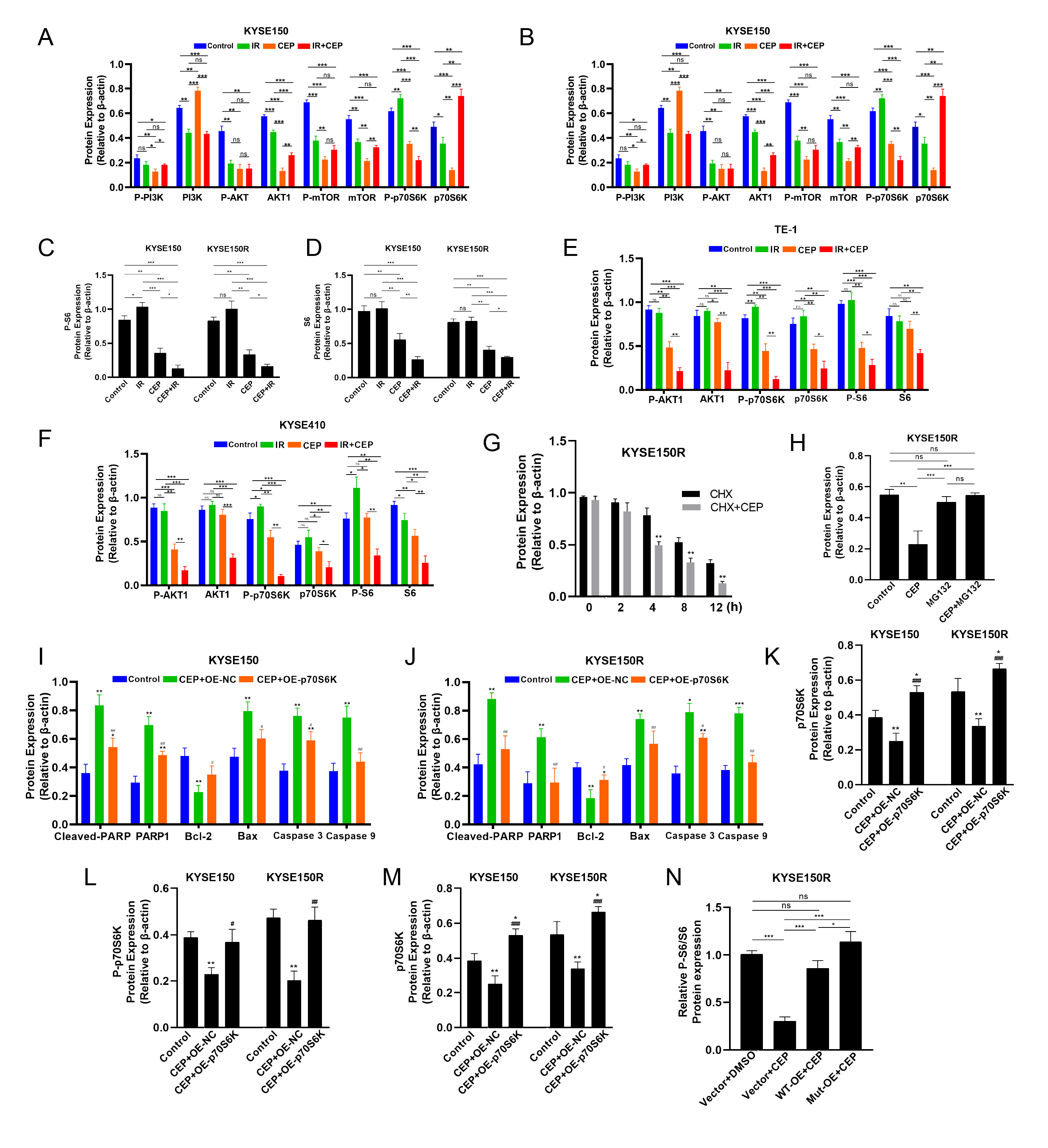


Figure S3. A-F Quantitative western blotting analysis of total and phosphorylated PI3K, AKT1, mTOR, p70S6K, and S6 in KYSE150, KYSE150R (A-D), TE-1, and KYSE410 (E-F) cells. G Quantitative analysis of p70S6K degradation kinetics from the CHX chase assay. H Quantitative analysis of relative p70S6K protein levels following the indicated treatments. I-M Quantitative western blotting analysis of p70S6K, P-p70S6K, and downstream apoptosis-related proteins in OE-NC and OE-p70S6K cells following CEP exposure. * *P* < 0.05, ** *P* < 0.01, *** *P* < 0.001 compared with the Control group; # *P* < 0.05, ## *P* < 0.01, ### *P* < 0.001 compared with the CEP+OE-NC group. N Quantitative analysis of the relative p-S6/S6 ratio across the indicated experimental groups. * *P* < 0.05, ** *P* < 0.01, *** *P* < 0.001; ns, not significant.


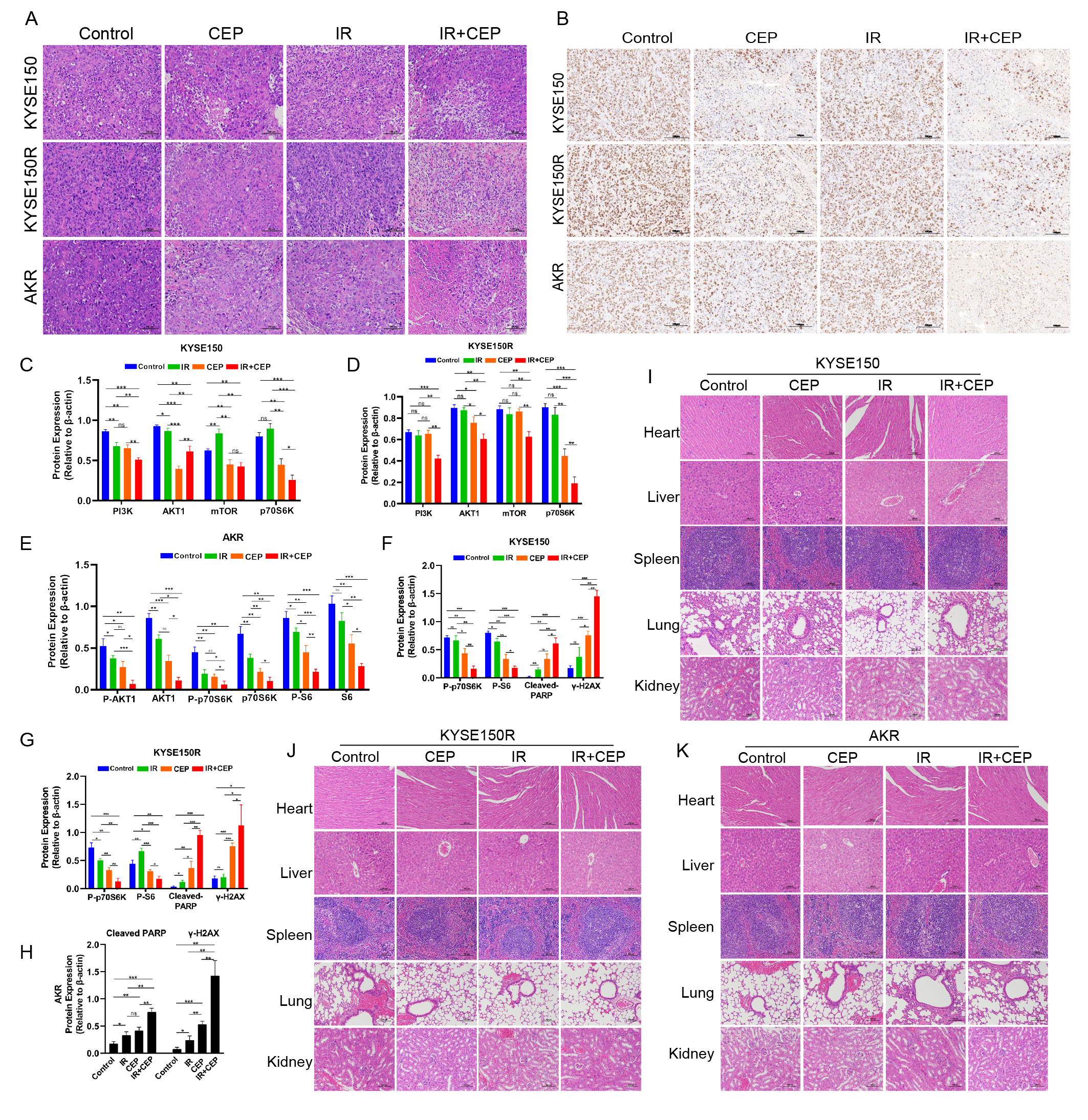


Figure S4. Histological analysis, protein quantification, and safety evaluation in vivo.

A Representative H&E staining images of tumor sections from KYSE150, KYSE150R, and AKR models across the indicated treatment groups. B Representative immunohistochemistry (IHC) images evaluating the expression of the proliferation marker Ki-67 in the corresponding tumor tissues. C-H Densitometric quantification of Western blot results showing the relative protein expression levels of the PI3K/Akt/mTOR/p70S6K signaling pathway components, DNA damage marker (γ-H2AX), and apoptosis marker (Cleaved-PARP) in tumor lysates from KYSE150 (C, F), KYSE150R (D, G), and AKR (E, H) models. I-K Representative H&E staining images of major vital organs (heart, liver, spleen, lungs, and kidneys) to assess in vivo systemic toxicity and safety. Data are presented as mean ± SD. **P* < 0.05, *P* < 0.01, ****P* < 0.001.

**Supplementary tables**

Table S1. Detailed information of 62 patients evaluated as partial response (PR) and stable disease (SD) after radiotherapy.

|  | Evaluation of radiotherapy | | *P* Value |
| --- | --- | --- | --- |
|  | Partial response  (PR, n=31) | Stable disease  (SD, n=31) |
| Sex |  |  | 0.793 |
| Male | 20 | 19 |  |
| Female | 11 | 12 |  |
| Primary tumor location |  |  | 0.590 |
| Upper | 13 | 11 |  |
| Middle | 12 | 12 |  |
| Lower | 6 | 8 |  |
| Stage |  |  | 0.127 |
| Ⅰ+Ⅱ | 10 | 4 |  |
| Ⅲ+Ⅳ | 21 | 27 |  |
| T Stage |  |  | 0.335 |
| T1~T2 | 8 | 4 |  |
| T3~T4 | 23 | 27 |  |
| Distance metastasis |  |  | 0.731 |
| M0 | 27 | 25 |  |
| M1 | 4 | 6 |  |

Table S2. Detailed information of 14 patients evaluated as PR or SD after radiotherapy.

|  | Evaluation of radiotherapy | |
| --- | --- | --- |
|  | Partial response  (PR, n=7) | Stable disease  (SD, n=7) |
| Sex |  |  |
| Male | 2 | 5 |
| Female | 5 | 2 |
| Primary tumor location | |  |
| Upper | 5 | 3 |
| Middle | 2 | 3 |
| Lower | 0 | 1 |
| Stage |  |  |
| Ⅰ+Ⅱ | 1 | 2 |
| Ⅲ+Ⅳ | 6 | 5 |
| T Stage |  |  |
| T1~T2 | 0 | 1 |
| T3~T4 | 7 | 6 |
| Distance metastasis |  |  |
| M0 | 6 | 6 |
| M1 | 1 | 1 |

Table S3. Detailed information of 34 patients evaluated as PR or SD after radiotherapy.

|  | Evaluation of radiotherapy | |  |
| --- | --- | --- | --- |
|  | Partial response  (PR, n=17) | Stable disease  (SD, n=17) | *P* Value |
| Sex |  |  | 0.473 |
| Male | 10 | 12 |  |
| Female | 7 | 5 |  |
| Primary tumor location | |  |  |
| Upper | 6 | 4 | 0.740 |
| Middle | 8 | 9 |  |
| Lower | 3 | 4 |  |
| Stage |  |  | 0.244 |
| Ⅰ+Ⅱ | 6 | 3 |  |
| Ⅲ+Ⅳ | 11 | 14 |  |
| T Stage |  |  |  |
| T1~T2 | 4 | 3 | 0.671 |
| T3~T4 | 13 | 14 |  |
| Distance metastasis |  |  |  |
| M0 | 15 | 14 | 0.628 |
| M1 | 2 | 3 |  |

Table S4. Consistent upregulation of core energy metabolites in radioresistant ESCC patients across initial screening and expanded validation cohorts.

| Pathway | Metabolite | Initial screening  (n=7) | Expanded Targeted Confirmation (n=17) | Trend |
| --- | --- | --- | --- | --- |
| Glycolysis | Pyruvic acid | *P*=0.002  FC=2.28 | *P*=0.009  FC=1.81 | Consistently upregulated |
| Glycolysis | Lactic acid | *P*＞0.05  FC=1.31 | *P*=0.022  FC=1.21 | Confirmed in expanded cohort |
| TCA Cycle | Citric acid | *P*＞0.05  FC=1.16 | *P*=0.022  FC=1.23 | Confirmed in expanded cohort |
| TCA Cycle | Oxoglutaric acid | *P*=0.024  FC=1.45 | *P*=0.001  FC=1.52 | Consistently upregulated |
| TCA Cycle | Succinic acid | *P*＞0.05  FC=1.48 | *P*=0.006  FC=1.37 | Confirmed in expanded cohort |
| TCA Cycle | Malic acid | *P*＞0.05  FC=1.20 | *P*=0.026  FC=1.32 | Confirmed in expanded cohort |

The table details the statistical significance (*P*-value) and fold change (FC) of key intermediates involved in glycolysis and the TCA cycle. Data are compared between radiosensitive (PR) and radioresistant (SD) patients across two independent analytical phases: the initial untargeted screening (n=7 pairs) and the expanded targeted confirmation (n=17 pairs). An FC > 1 indicates upregulation in the radioresistant group. Metabolites lacking statistical significance in the initial small-scale screening (*P* > 0.05) still demonstrated a consistent upward trend (FC > 1), which was subsequently validated in the expanded cohort.

Table S5. Detailed information of 10 ESCC patients who conducted [immunohistochemistry](javascript:;) (IHC) experiments.

|  | Number of patients |
| --- | --- |
| Sex |  |
| Male | 6 |
| Female | 4 |
| Primary tumor location |  |
| Upper | 2 |
| Middle | 5 |
| Lower | 3 |
| Stage |  |
| Ⅱ | 6 |
| Ⅲ | 3 |
| Ⅳ | 1 |
| T Stage |  |
| T1,T2 | 1 |
| T3 | 9 |
| Lymph node metastasis |  |
| Absent | 2 |
| Present | 8 |
| Distance metastasis |  |
| M0 | 9 |
| M1 | 1 |

**Table S6** Primer sequencesPrimer sequences of reverse transcription-quantiative PCR reactions

P70S6K-Q347A:

| **Name** | **Direction** | **Sequence (5’-3’)** |
| --- | --- | --- |
| HIF-1α | Forward primer | GAACGTCGAAAAGAAAAGTCTCG |
|  | Reverse primer | CCTTATCAAGATGCGAACTCACA |
| HK1 | Forward primer | ATGGCTGCGGTTGTGGATAA |
|  | Reverse primer | CTGCTTGCCTCTGTGCGTAA |
| HK2 | Forward primer | TTGGAGCCACCACTCACCCTA |
|  | Reverse primer | GAGCCCATTGTCCGTTACTTTC |
| LDHA | Forward primer | CGTGGCTTGGAAGATAAGTGGT |
|  | Reverse primer | CAGAGAGACACCAGCAACATTCA |
| GAPDH | Forward primer | GGAAGCTTGTCATCAATGGAAATC |
|  | Reverse primer | TGATGACCCTTTTGGCTCCC |
| UQCRQ | Forward primer | GCCTATCCGCACGTCTTCACT |
|  | Reverse primer | ACACTACAAACTGCGGCACCAC |
| PKM2 | Forward primer | GCTGCCATCTACCACTTGCA |
|  | Reverse primer | CCAGACTTGGTGAGGACGAT |
| PFK1 | Forward primer | CAGCTTGCGTCGTGTCACTG |
|  | Reverse primer | TGATGGCAAGTCGCTTGTAGG |
| IDH3B | Forward primer | GGTGGGAGATCCTCAGGTCA |
|  | Reverse primer | AAACCGCAACCGAACTACC |
| SDHA | Forward primer | CAAACAGGAACCCGAGGTTTT |
|  | Reverse primer | CAGCTTGGTAACACATGCTGT |
| SDHB | Forward primer | AATTTGCCATCTACCGAATGC |
|  | Reverse primer | GCGTCTCGATGCTCAGAATG |
| TOMM20 | Forward primer | AGGTGAATCTTGGCTTGCAG |
|  | Reverse primer | GTGCTGCTATCCAGGTAACC |
| NQO1 | Forward primer | CGCAGACCTTGTGATATTCCAG |
|  | Reverse primer | CGTTTCTTCCATCCTTCCAGG |

ATGAGGCGACGAAGGAGGCGGGACGGCTTTTACCCAGCCCCGGACTTCCGAGACAGGGAAGCTGAGGACATGGCAGGAGTGTTTGACATAGACCTGGACCAGCCAGAGGACGCGGGCTCTGAGGATGAGCTGGAGGAGGGGGGTCAGTTAAATGAAAGCATGGACCATGGGGGAGTTGGACCATATGAACTTGGCATGGAACATTGTGAGAAATTTGAAATCTCAGAAACTAGTGTGAACAGAGGGCCAGAAAAAATCAGACCAGAATGTTTTGAGCTACTTCGGGTACTTGGTAAAGGGGGCTATGGAAAGGTTTTTCAAGTACGAAAAGTAACAGGAGCAAATACTGGGAAAATATTTGCCATGAAGGTGCTTAAAAAGGCAATGATAGTAAGAAATGCTAAAGATACAGCTCATACAAAAGCAGAACGGAATATTCTGGAGGAAGTAAAGCATCCCTTCATCGTGGATTTAATTTATGCCTTTCAGACTGGTGGAAAACTCTACCTCATCCTTGAGTATCTCAGTGGAGGAGAACTATTTATGCAGTTAGAAAGAGAGGGAATATTTATGGAAGACACTGCCTGCTTTTACTTGGCAGAAATCTCCATGGCTTTGGGGCATTTACATCAAAAGGGGATCATCTACAGAGACCTGAAGCCGGAGAATATCATGCTTAATCACCAAGGTCATGTGAAACTAACAGACTTTGGACTATGCAAAGAATCTATTCATGATGGAACAGTCACACACACATTTTGTGGAACAATAGAATACATGGCCCCTGAAATCTTGATGAGAAGTGGCCACAATCGTGCTGTGGATTGGTGGAGTTTGGGAGCATTAATGTATGACATGCTGACTGGAGCACCCCCATTCACTGGGGAGAATAGAAAGAAAACAATTGACAAAATCCTCAAATGTAAACTCAATTTGCCTCCCTACCTCACACAAGAAGCCAGAGATCTGCTTAAAAAGCTGCTGAAAAGAAATGCTGCTTCTCGTCTGGGAGCTGGTCCTGGGGACGCTGGAGAAGTTgcaGCTCATCCATTCTTTAGACACATTAACTGGGAAGAACTTCTGGCTCGAAAGGTGGAGCCCCCCTTTAAACCTCTGTTGCAATCTGAAGAGGATGTAAGTCAGTTTGATTCCAAGTTTACACGTCAGACACCTGTCGACAGCCCAGATGACTCAACTCTCAGTGAAAGTGCCAATCAGGTCTTTCTGGGTTTTACATATGTGGCTCCATCTGTACTTGAAAGTGTGAAAGAAAAGTTTTCCTTTGAACCAAAAATCCGATCACCTCGAAGATTTATTGGCAGCCCACGAACACCTGTCAGCCCAGTCAAATTTTCTCCTGGGGATTTCTGGGGAAGAGGTGCTTCGGCCAGCACAGCAAATCCTCAGACACCTGTGGAATACCCAATGGAAACAAGTGGCATAGAGCAGATGGATGTGACAATGAGTGGGGAAGCATCGGCACCACTTCCAATACGACAGCCGAACTCTGGGCCATACAAAAAACAAGCTTTTCCCATGATCTCCAAACGGCCAGAGCACCTGCGTATGAATCTATGA
