# Supplementary figures and images for: Repurposing cepharanthine as a radiosensitizer in esophageal squamous cell carcinoma through dual metabolic intervention and direct targeting of p70s6K
Source: J Transl Med. 2026 Jul 3;24:893. doi: 10.1186/s12967-026-08550-y (PMC13359829; doi:10.1186/s12967-026-08550-y)

**Figure 2**

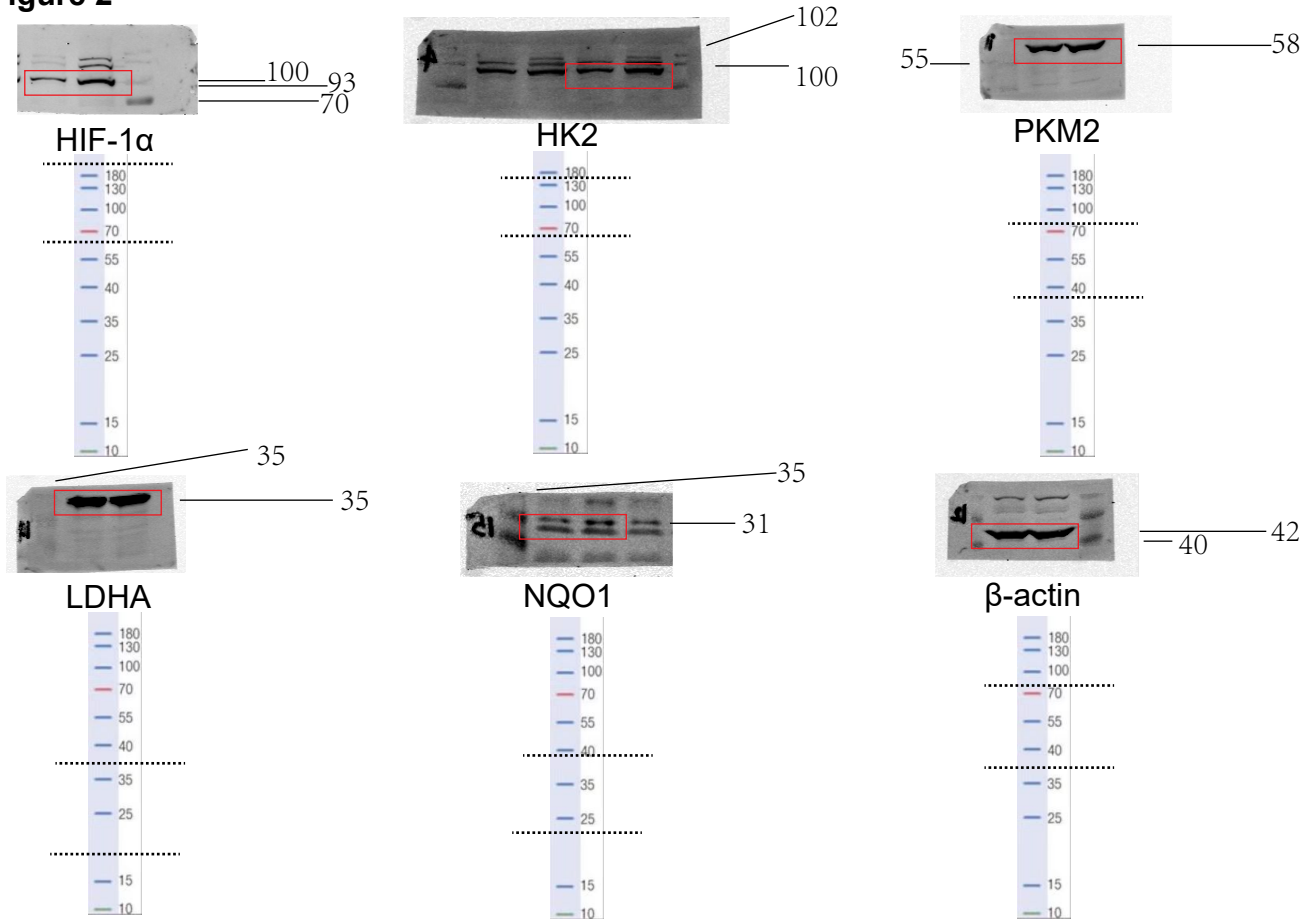

**Figure 4**

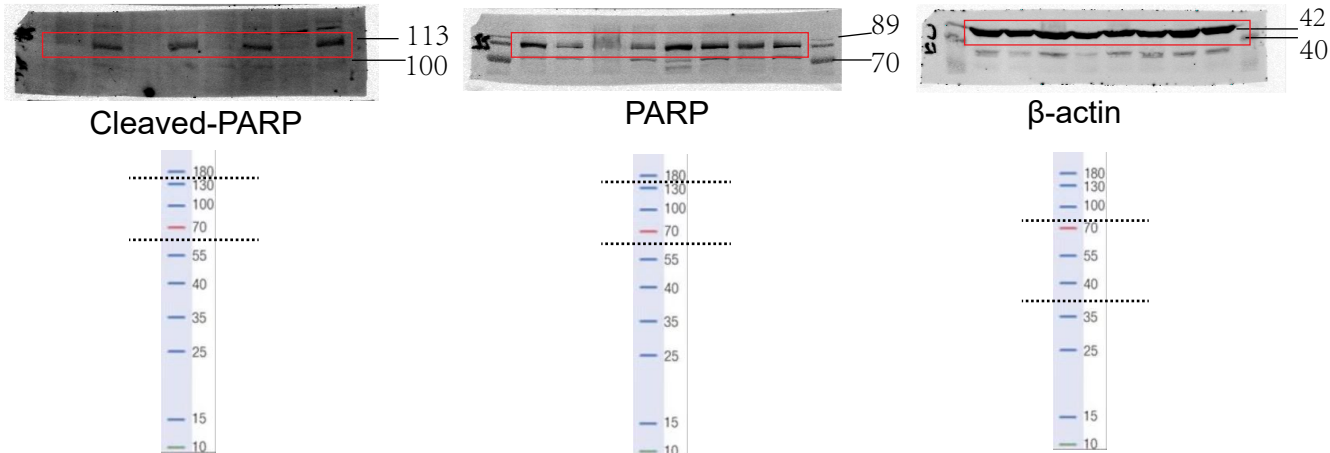

**Figure 4**

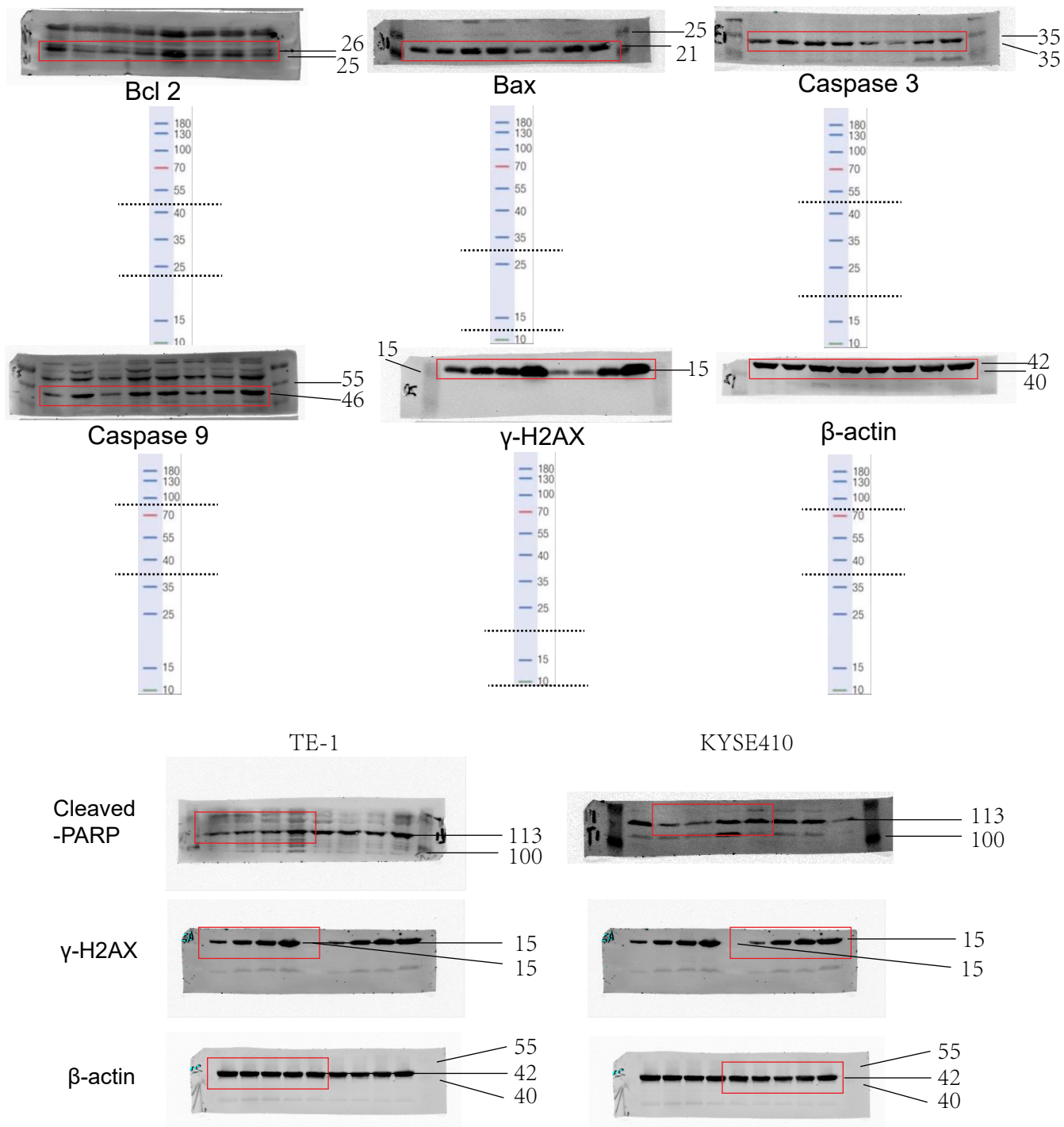

**Figure 5**

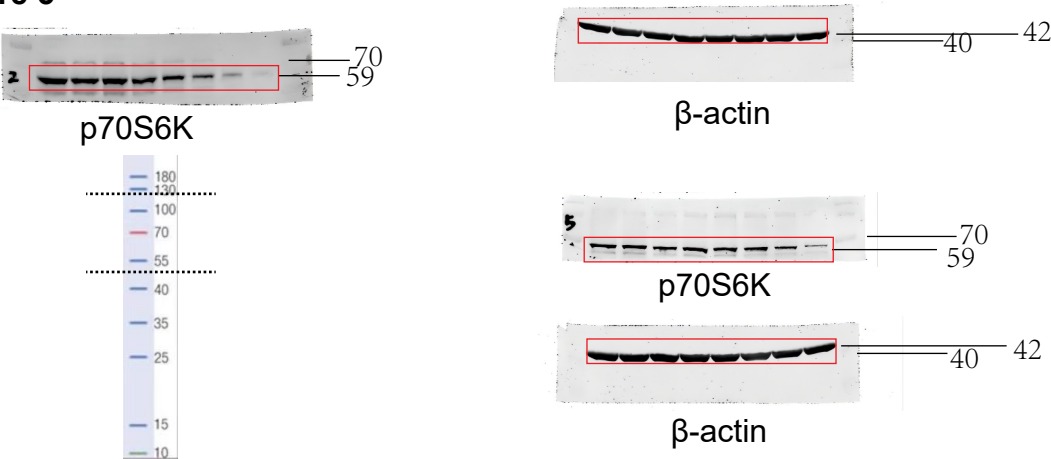

**Figure 6**

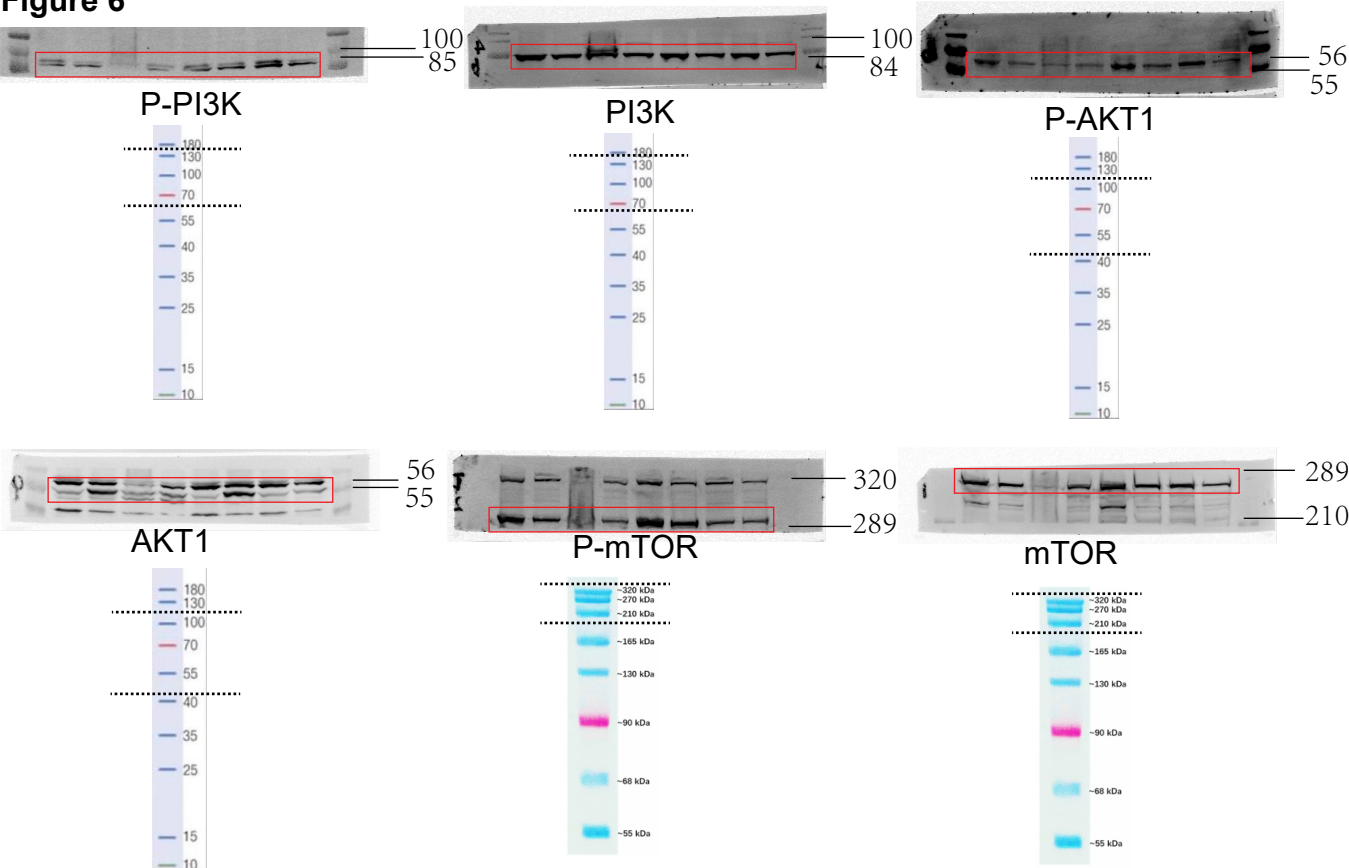

Figure 6

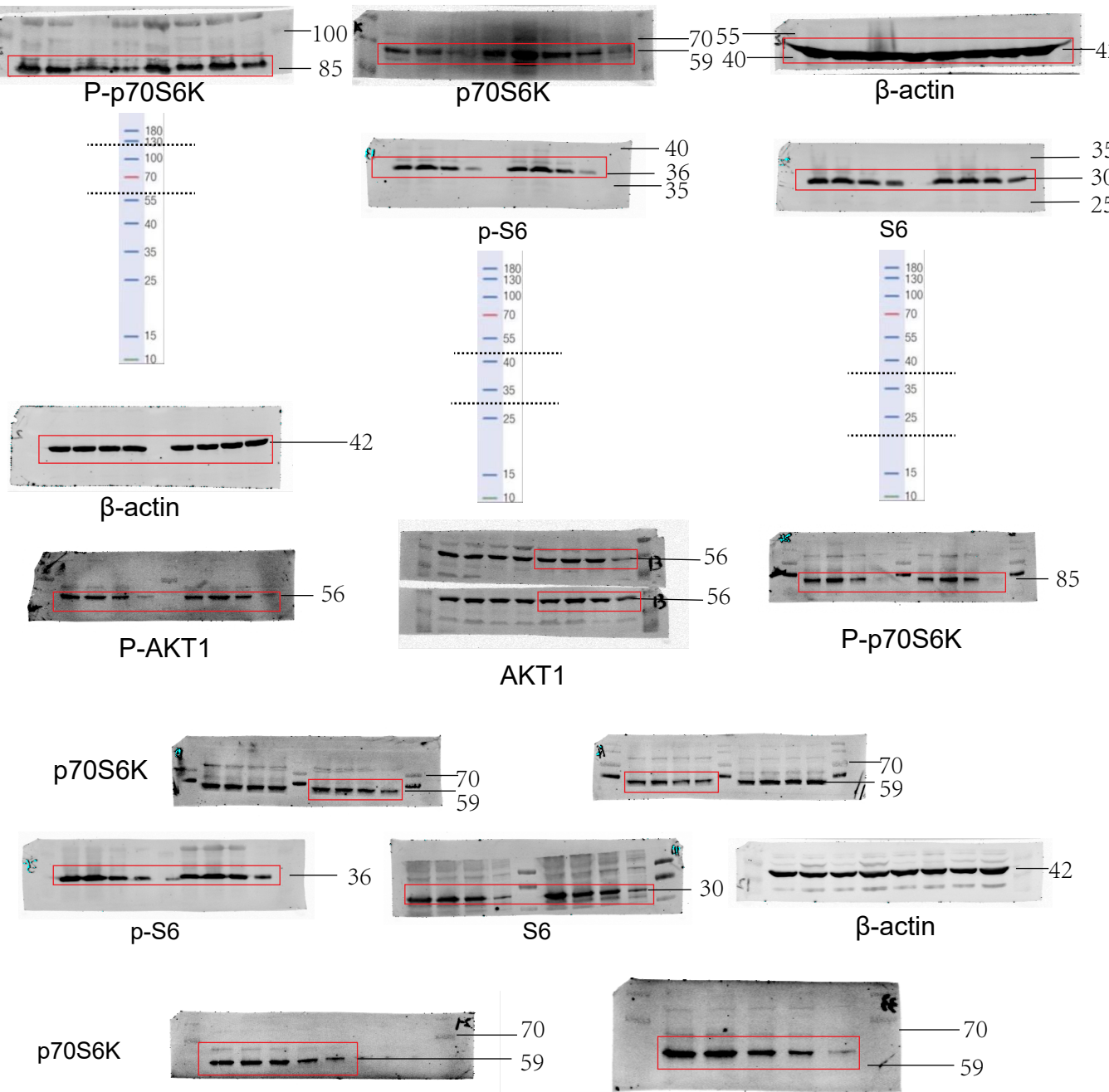

**Figure 6**

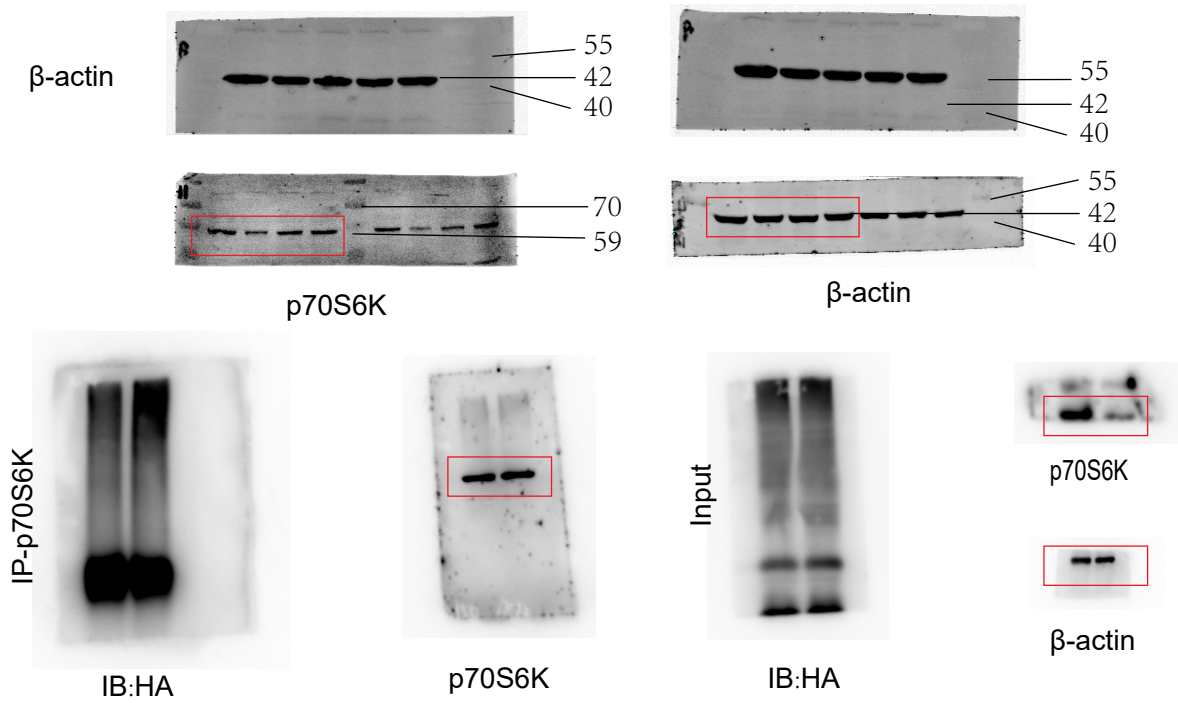

**Figure 7**

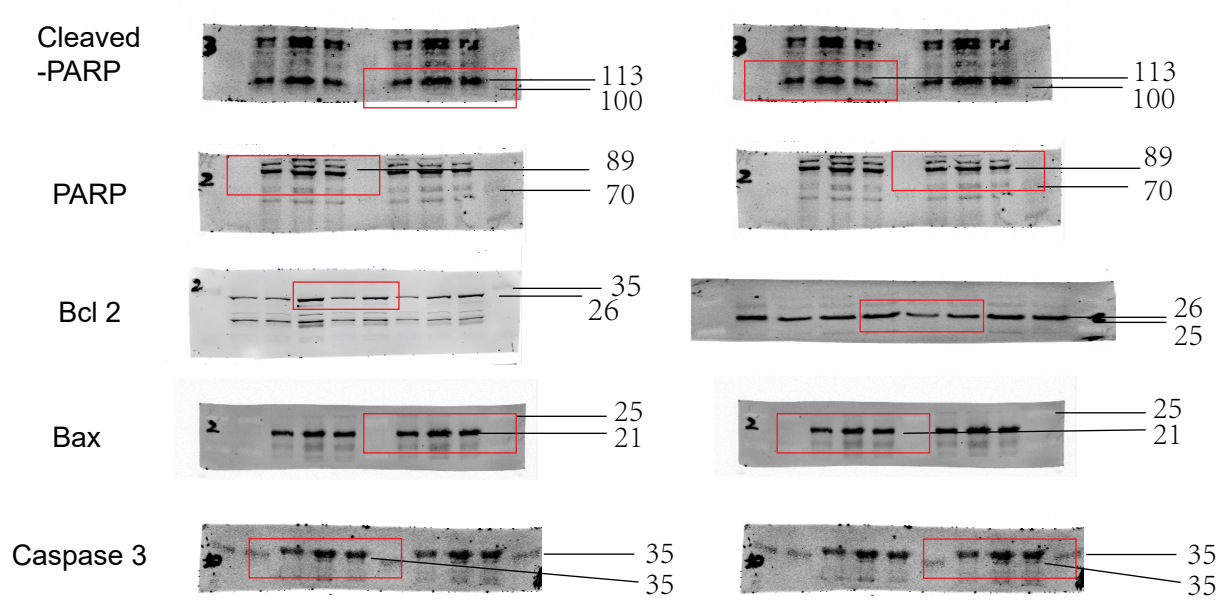

**Figure 7**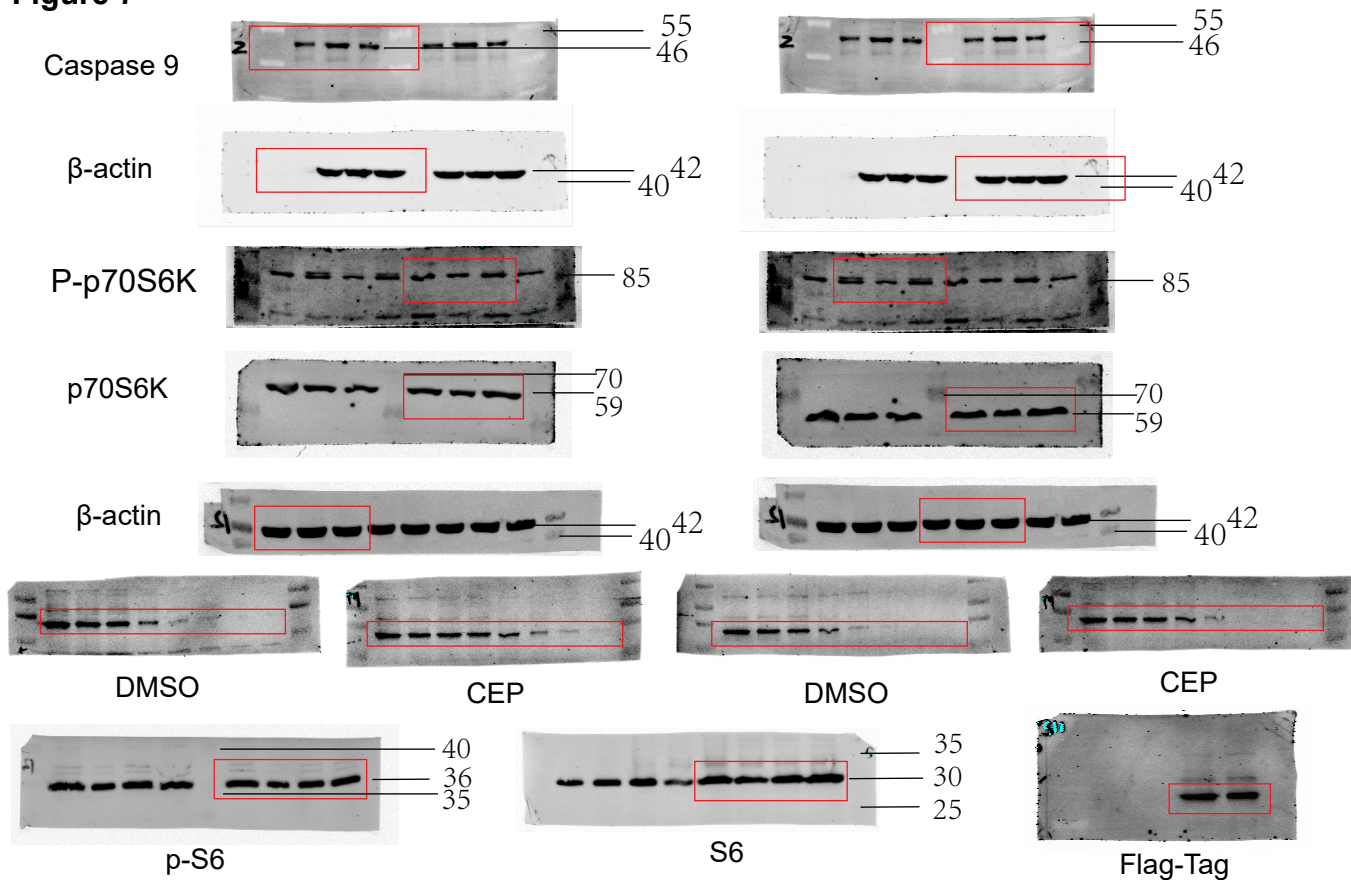**Figure 8**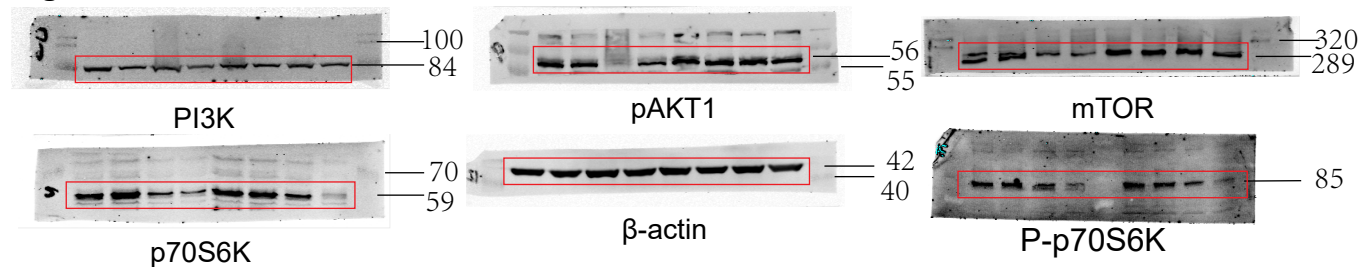

**Figure 8**

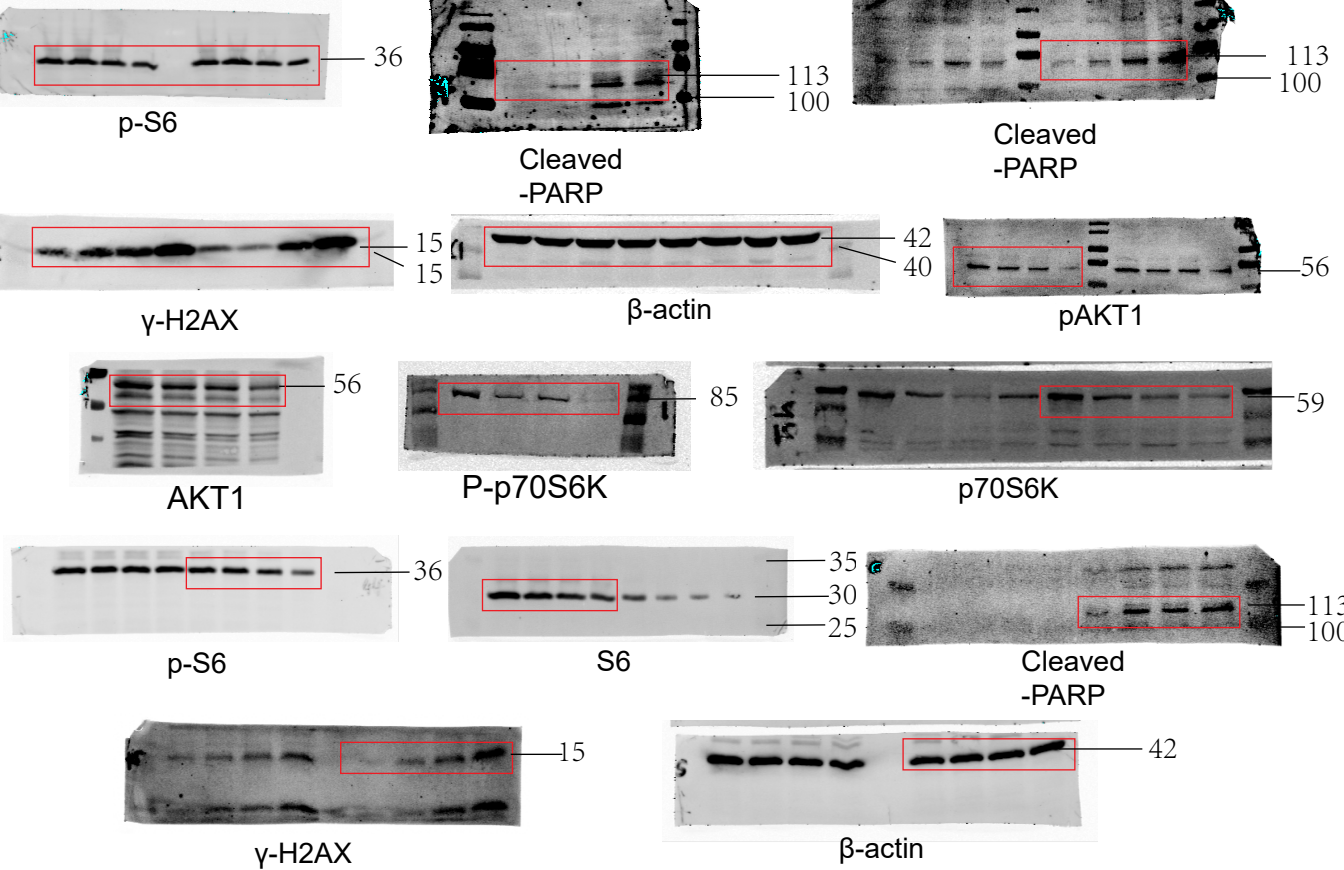

Supplement: Supplementary file 2 — Supplementary Material 2 [file 12967_2026_8550_MOESM2_ESM.pdf]
